# Supplementary material for: Prescription of benzodiazepines, z-drugs, and gabapentinoids and mortality risk in people receiving opioid agonist treatment: Observational study based on the UK Clinical Practice Research Datalink and Office for National Statistics death records
Source: PLoS Med. 2019 Nov 26;16(11):e1002965. doi: 10.1371/journal.pmed.1002965 (PMC6879111; doi:10.1371/journal.pmed.1002965)
Supplement: S1 Appendix — (DOCX) [file pmed.1002965.s001.docx]

**Appendix. Summary of analyses and associated commands in Stata.**

First, Cox proportional hazards models were used to assess survival in relation to co-prescription (the exposure):

stcox exposure (unadjusted analyses, table 3)

We then also adjusted for age, gender, year, comorbidity, region, OST type, OST treatment period, and where applicable benzodiazepine, z-drug and gabapentinoid exposure, by adding all these variables as covariates into the survival model. Some covariates were constant across each individual (e.g. gender, region), and others varied by episode (age, year, comorbidity) and within episodes (OST type, OST treatment period, other prescibed drugs).

stcox exposure covariates (adjusted analyses, table 3)

Where there was evidence of an association between the exposure and survival, we investigated whether the association depended on the OST treatment period. We fitted an interaction, allowing the effect of exposure to differ across the two categories of treatment (on or off treatment).

stcox exposure*treatment period (Analyses in Table 4)

We then examined the association between concurrent prescription and survival, by including as an exposure only benzodiazepine that was prescribed during the period on OST treatment.

stcox exposure covariates (adjusted analyses, table 5)
