# Supplementary material for: Prescription of benzodiazepines, z-drugs, and gabapentinoids and mortality risk in people receiving opioid agonist treatment: Observational study based on the UK Clinical Practice Research Datalink and Office for National Statistics death records
Source: PLoS Med. 2019 Nov 26;16(11):e1002965. doi: 10.1371/journal.pmed.1002965 (PMC6879111; doi:10.1371/journal.pmed.1002965)
Supplement: S1 Patient Flowchart — (DOCX) [file pmed.1002965.s002.docx]

**Patient flow-chart**


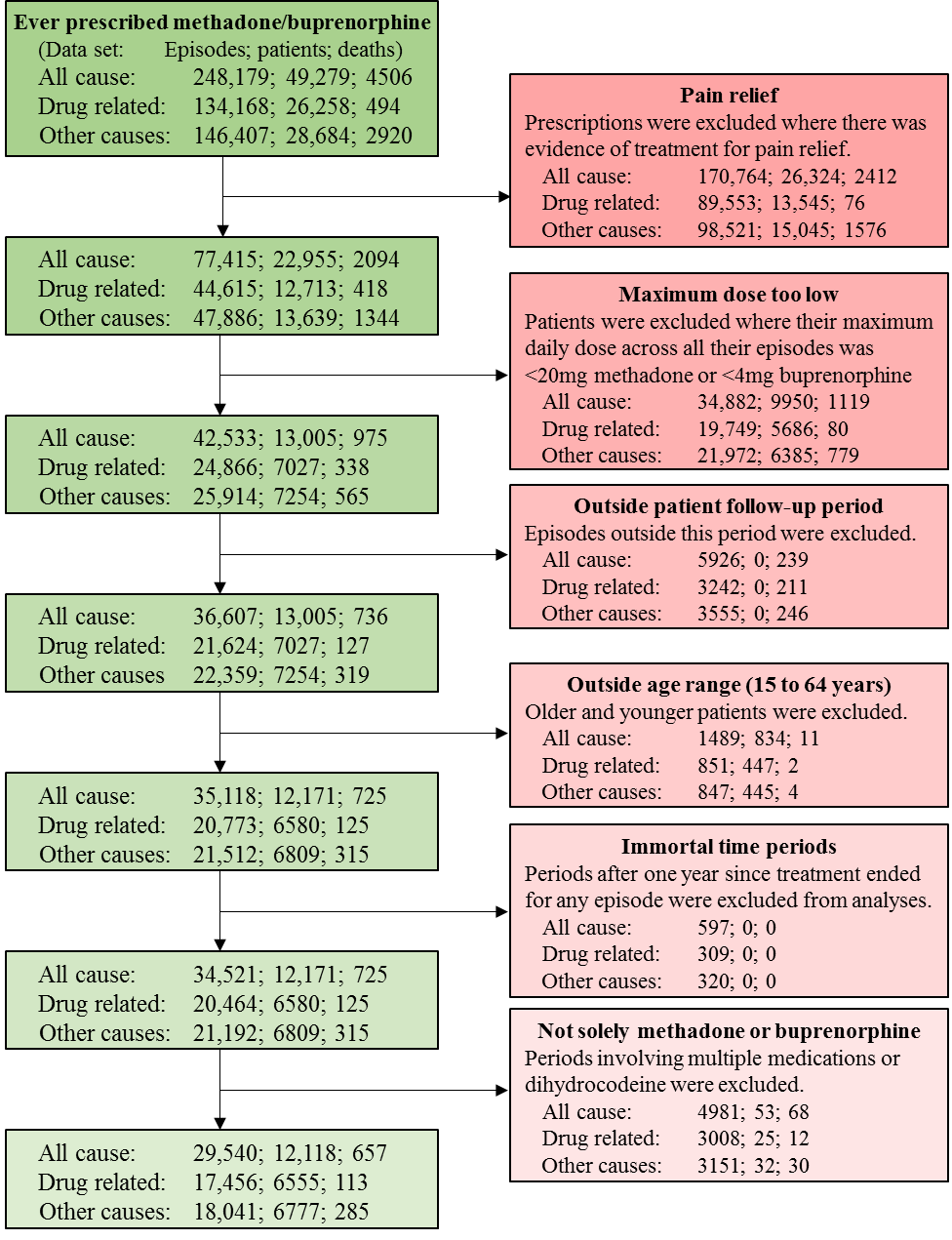


The above figure shows numbers of episodes, patients and deaths for the all-cause, drug related and other non-drug related data sets. Identification of prescriptions for pain relief were based upon prescription text, medication in the form of patches or episodes of dihydrocodeine prior to starting OST. The follow-up period varied by patient and reflected a combination of the study period (January 1998 to July 2014), the patient registration period with the primary care practice, the CPRD usable data date and one year after the last treatment ended. The exclusion of periods greater than one year after the cessation of treatment for each episode affected the person-years at risk but not the number of deaths. The final number of patients for drug related and other causes includes 216 and 266 patients who were known to have died from ONS records but this death occurred outside the patient follow-up periods. This implies that there were 6226 patients with no recorded death in ONS records which were included in both these data sets.
