# Supplementary material for: Prescription of benzodiazepines, z-drugs, and gabapentinoids and mortality risk in people receiving opioid agonist treatment: Observational study based on the UK Clinical Practice Research Datalink and Office for National Statistics death records
Source: PLoS Med. 2019 Nov 26;16(11):e1002965. doi: 10.1371/journal.pmed.1002965 (PMC6879111; doi:10.1371/journal.pmed.1002965)
Supplement: S1 Protocol — (DOCX) [file pmed.1002965.s003.docx]

**ISAC APPLICATION FORM**

**PROTOCOLS FOR RESEARCH USING THE CLINICAL PRACTICE RESEARCH DATALINK (CPRD)**

| ISAC use only:  Protocol Number  Date submitted | 14_073R2Mn2  09/12/2015 | **IMPORTANT**  **If you have any queries, please contact ISAC Secretariat:** ISAC[@cprd.com](mailto:Annalisa.Rubino@gprd.com) |
| --- | --- | --- |

| 1. Study Title 2. Evaluating the impact of opiate substitution treatment on drug related deaths in the population: a natural experiment using primary care, other drug treatment databases & model projections |
| --- |
| 1. Principal Investigator (full name, job title, organisation & e-mail address for correspondence regarding this protocol)   Matt Hickman, Professor of Public Health and Epidemiology,  Tel: 0117 928 7252  E-mail: matthew.hickman@bristol.ac.uk |
| 1. Affiliation (full address)   School of Social and Community Medicine, University of Bristol, Canynge Hall, 39 Whatley Road, Bristol BS8 2PS |
| 1. Protocol’s Author (if different from the principal investigator) |
| 1. List of all investigators/collaborators (*please list the names, affiliations and e-mail addresses* of all collaborators*, other than the principal investigator)   Professor John Macleod University of Bristol: john.macleod@bristol.ac.uk  Professor John Strang Institute of Psychiatry: john.strang@kcl.ac.uk  Dr Tim Millar University of Manchester: tim.millar@manchester.ac.uk  Mr Ken Stringer M-alliance: ken@m-alliance.org.uk  Dr John Marsden Institute of Psychiatry : john.marsden@kcl.ac.uk  Professor Kate Tilling University of Bristol: kate.tilling@bristol.ac.uk  Dr Peter Vickerman, University of Bristol: peter.vickerman@bristol.ac.uk  Ms Barbara Coleman Bristol City Council:barbara.coleman@bristol.gov.uk  Dr Colin Steer, University of Bristol: Colin.Steer@bristol.ac.uk  **Please note that your ISAC application form and protocol* ***must*** *be copied to all e-mail addresses listed above at the time of submission of your application to the ISAC mailbox. Failure to do so will result in delays in the processing of your application.* |
| 1. Type of Institution (please tick one box below)   Academia **x** Research Service Provider  Pharmaceutical Industry  NHS  Government Departments  Others |
| 1. Financial Sponsor of study   Pharmaceutical Industry *(please specify)*        Academia*(please specify)*  Government / NHS *(please specify)* x NIHR None  Other *(please specify)* |
| 1. Data source *(please tick one box below)*   Sponsor has on-line access  Purchase of ad hoc dataset  Commissioned study  Other x *(please specify)* Also NIHR School of Public Health – privileged access |
| 1. Has this protocol been peer reviewed by another Committee?   Yes* xNo  ** Please state in your protocol the name of the reviewing Committee(s) and provide an outline of the review process and outcome. NIHR Health Services and Delivery Research Programme* |
| 1. Type of Study *(please tick all the relevant boxes which apply)*   Adverse Drug Reaction/Drug Safety Drug Use  Disease Epidemiology x  Drug Effectiveness  Pharmacoeconomic  Other |
| 1. This study is intended for:   Publication in peer reviewed journals x Presentation at scientific conference x  Presentation at company/institutional meetings  Other: Expert invited Meetings at UK Government and European Scientific Meetings |

| 1. Does this protocol also seek access to data held under the CPRD Data Linkage Scheme?   Yes x No |
| --- |
| 1. If you are seeking access to data held under the CPRD Data Linkage Scheme*, please select the source(s) of linked data being requested.   Hospital Episode Statistics  Cancer Registry Data**  MINAP x ONS Mortality Data  Index of Multiple Deprivation/ Townsend Score  Mother Baby Link  Other: (please specify)  ** As part of the ISAC review of linkages, the protocol may be shared - in confidence - with a representative of the requested linked data set(s) and summary details may be shared - in confidence - with the Confidentiality Advisory Group of the Health Research Authority.*  ***Please note that applicants seeking access to cancer registry data must provide consent for publication of their study title and study institution on the UK Cancer Registry website. Please contact the CPRD Research Team on +44 (20) 3080 6383 or email* [*kc@cprd.com*](mailto:kc@cprd.com) *to discuss this requirement further.* |
| 1. If you are seeking access to data held under the CPRD Data Linkage Scheme, have you already discussed your request with a member of the Research team?   Yes x No*  **Please contact the CPRD Research Team on +44 (20) 3080 6383 or email* [*kc@cprd.com*](mailto:kc@cprd.com) *to discuss your requirements before submitting your application.*  Please list below the name of the person/s at the CPRD with whom you have discussed your request.  Daniel Dedman |
| 1. If you are seeking access to data held under the CPRD Data Linkage Scheme, please provide the following information:   The number of linked datasets requested: CPRD with ONS mortality register  A synopsis of the purpose(s) for which the linkages are required:  Death certificate/ cause of death information      Is linkage to a local dataset with <1 million patients being requested?  Yes*  No x  ** If yes, please provide further details:* |
| 1. If you have requested linked data sets, please indicate whether the Principal Investigator or any of the collaborators listed in response to question 5 above, have access to any of the linked datasets in a patient identifiable form, or associated with a patient index.   Yes*  No x  ** If yes, please provide further details:* |
| 1. Does this protocol involve requesting any additional information from GPs?   Yes*  No x  * *Please indicate what will be required:*  Completion of questionnaires by the GP*^ψ^* Yes  No  Provision of anonymised records (e.g. hospital discharge summaries) Yes  No  Other (please describe)  *^ψ^ Any questionnaire for completion by GPs or other health care professional must be approved by ISAC before circulation for completion.* |
| 1. Does this protocol describe a purely observational study using CPRD data (this may include the review of anonymised free text)?   Yes* x No**  ** Yes: If you will be using data obtained from the CPRD Group, this study does not require separate ethics approval from an NHS Research Ethics Committee.*  *** No: You may need to seek separate ethics approval from an NHS Research Ethics Committee for this study. The ISAC will provide advice on whether this may be needed.* |
| 1. Does this study involve linking to patient *identifiable* data from other sources?   Yes  No x |
| 1. Does this study require contact with patients in order for them to complete a questionnaire?   Yes  No x  *N.B. Any questionnaire for completion by patients must be approved by ISAC before circulation for completion.* |
| 1. Does this study require contact with patients in order to collect a sample?   Yes*  No x  ** Please state what will be collected* |

| 1. Experience/expertise available   Please complete the following questions to indicate the experience/expertise available within the team of researchers actively involved in the proposed research, including analysis of data and interpretation of results |
| --- |
| Previous GPRD/CPRD Studies Publications using GPRD/CPRD data  None  1-3 x x  > 3 |
| Yes No  Is statistical expertise available within the research team? x  *If yes, please outline level of experience* *Professor of Medical Statistics*  *And RA in statistics*  Is experience of handling large data sets (>1 million records)  available within the research team? x  *If yes, please outline level of experience* *Previous GPRD experience*  Is UK primary care experience available within the research team? x  *If yes, please outline level of experience* *Professor of Primary Care Epidemiology & Link to NIHR School of Primary Care Research* |
| 1. References relating to your study   Please list up to 3 references (most relevant) relating to your proposed study.  Cornish R, Macleod J, Strang J, Vickerman P, Hickman M. Risk of death during and after opiate substitution treatment in primary care: prospective observational study in UK general practice research database. BMJ 2010;341:c5475.  Degenhardt L, Randall D, Hall W, Law M, Butler T, Burns L. Mortality among clients of a state-wide opioid pharmacotherapy program over 20 years: risk factors and lives saved. Drug Alcohol Depend 2009 November 1;105(1-2):9-15.  McCowan C, Kidd B, Fahey T. Factors associated with mortality in Scottish patients receiving methadone in primary care: retrospective cohort study. BMJ 2009;338(doi: 10.1136/bmj.b2225.). |

**Protocol content checklist**

In order to help ensure that protocols submitted for review contain adequate information for protocol evaluation, ISAC have produced instructions on the content of protocols for research using CPRD data. These instructions are available on the CPRD website ([www.cprd.com/ISAC](http://www.cprd.com/ISAC)). All protocols using CPRD data which are submitted for review by ISAC must contain information on the areas detailed in the instructions. IF you do not feel that a specific area required by ISAC is relevant for your protocol, you will need to justify this decision to ISAC.

Applicants must complete the checklist below to confirm that the protocol being submitted includes all the areas required by ISAC, or to provide justification where a required area is not considered to be relevant for a specific protocol. Protocols will not be circulated to ISAC for review until the checklist has been completed by the applicant.

**Please note, your protocol will be returned to you if you do not complete this checklist, or if you answer ‘no’ and fail to include justification for the omission of any required area.**

|  | **Included in protocol?** | |  |
| --- | --- | --- | --- |
| Required area | Yes | No | If no, reason for omission |
| *Lay Summary (max.200 words)* | x |  |  |
| *Background* | x |  |  |
| *Objective, specific aims and rationale* | x |  |  |
| *Study Type*  *Descriptive*  *Hypothesis Generating*  *Hypothesis Testing* | x  x  x |  |  |
| *Study Design* | x |  |  |
| *Sample size/power calculation*  *(Please provide justification of*  *sample size in the protocol)* | x |  |  |
| *Study population*  *(including estimate of expected number of*  *relevant patients in the CPRD)* | x |  |  |
| *Selection of comparison group(s) or controls* | x |  |  |
| *Exposures, outcomes and covariates*  *Exposures are clearly described*  *Outcomes are clearly described* | x  x  x |  |  |
| *Use of linked data*  *(if applicable)* | x |  |  |
| *Data/ Statistical Analysis Plan*  *There is plan for addressing confounding*  *There is a plan for addressing missing data* | x  x  x |  |  |
| *Patient/ user group involvement ^†^* | x |  |  |
| *Limitations of the study design, data sources*  *and analytic methods* | x |  |  |
| *Plans for disseminating and communicating study results* | x |  |  |

***^†^ It is expected that many studies will benefit from the involvement of patient or user groups in their planning and refinement, and/or in the interpretation of the results and plans for further work. This is particularly, but not exclusively true of studies with interests in the impact on quality of life. Please indicate whether or not you intend to engage patients in any of the ways mentioned above.***

***Voluntary registration of ISAC approved studies:***

*Epidemiological studies are increasingly being included in registries of research around the world, including those primarily set up for clinical trials. To increase awareness amongst researchers of ongoing research, ISAC encourages voluntary registration of epidemiological research conducted using MHRA databases. This will not replace information on ISAC approved protocols that may be published in its summary minutes or annual report. It is for the applicant to determine the most appropriate registry for their study. Please inform the ISAC secretariat that you have registered a protocol and provide the location.*

**HS&DR Project: 12/136/105** - Evaluating the impact of opiate substitution treatment on drug related deaths in the population: a natural experiment using primary care, other drug treatment databases & model projections: **ISAC CPRD Protocol 14_073R2** (AMENDMENT 27/10/2014)

**Lay Summary**

People who inject drugs (PWID) such as heroin have a risk of death >10 times higher than the general population. Overdose is the most common cause of death among PWID. The most effective treatment for heroin injectors is the prescription of legal, non-injected substitute drugs, most commonly methadone or buprenorphine. This is called opiate substitution treatment (OST) and in the UK is delivered mainly in primary care. Several recent studies have highlighted that there is a period of very high mortality risk in the first few months immediately after treatment cessation. In the UK OST prescription has expanded 5-fold in the last 10 years, but we have not observed any commensurate decrease in the number of drug related deaths in the population. This apparent paradox motivates our research i.e could the benefits of drug treatment be outweighed or balanced out by other factors which increase the risk of drug related deaths during or after OST? We will conduct a “natural experiment” and test our hypothesis by analysing data of people on OST and other drug treatments in the community and model how changes in the way drug treatment is delivered could reduce drug related deaths in the population.

**Background**

Opiate substitution treatment (OST) is the key treatment for heroin dependence and has been shown to have multiple benefits, including reductions in drug related crime, blood borne virus transmission, and improvements in social functioning(1-5). Prescribed OST in the community includes methadone, buprenorphine, and dihydrocodeine(1, 6). There are approximately 200,000 people who inject drugs (PWID), half exposed to drug treatment annually, mainly OST (75%) but also non-pharmacological treatments delivered in specialist drug agencies and residential units. OST is effective because it reduces heroin injection and is cost effective because of reductions in drug related crime and health harms(7). Observational studies have shown that the risk of mortality is reduced during opiate substitute treatment(8-12). Buprenorphine has been shown to be as good as methadone at treatment retention(13), and hypothetically is less likely to cause fatal overdose than methadone(14). In France, ecological analyses suggest that trends in overdose deaths are negatively associated with increases in buprenorphine prescription(15). However, direct comparative evidence on the risk of death during buprenorphine vs. methadone is yet to emerge.

Several recent studies also have highlighted that there is a period of very high mortality risk (>8 times higher than mortality risk during treatment) in the first few months immediately after treatment cessation (9-11, 16). In the UK analyses of primary care information have suggested that the risk of death in those who receive OST is twice as high among men compared to women, and is raised at the beginning and end of treatment, and maybe higher in those co-prescribed benzodiazepines(11, 17). In Australia the risk of death at treatment onset was greater than in the UK, with some evidence to suggest that the mortality risk at treatment onset was lower among those initiated onto buprenorphine compared to methadone, but these benefits may be offset by a shorter duration of treatment for those on buprenorphine compared to methadone(9). The evidence base on other drug treatments (residential rehabilitation, detoxification, and psychological treatments) is more limited; but studies also suggest that the risk of mortality is reduced during treatment and elevated after treatment (10, 18).

In the UK OST has expanded 5-fold in the last 10 years to over 1800kg per year (roughly equivalent to 33.3 million doses per year)(19). The rate of methadone deaths per gram of methadone prescribed has fallen, coinciding and attributed to the issuing and implementation of clinical guidelines recommending greater supervised consumption(20), but trends in the overall number of opiate related deaths have not declined (21) (22). Therefore, we aim to examine in more detail the risk of death during and after OST and other drug treatment in order to assess how changing the delivery of drug treatment could reduce the number of drug related deaths in the population.

**Objective, specific aims and rationale**

Our over-arching hypotheses are that the impact of opiate substitution treatment (OST) and other drug treatments in reducing drug related death (DRD) is influenced by modifiable treatment related factors (such as treatment duration, co-prescription of other drugs, treatment modality and dose, and dispensing arrangements); and that currently the benefits of OST and other non-pharmacological treatments in reducing drug related deaths in the population maybe outweighed or balanced out by other factors which increase risk of mortality.

The aim of the study, therefore, is to corroborate or refute these hypotheses in order to inform robust, credible evidence based treatment guidelines that can underpin the effective reduction of population drug-related deaths through primary care based intervention. This project will focus on the analysis of OST delivered in primary care through the analysis data from the Clinical Practice Research Datalink (a parallel project will analyse non-pharmacological treatments reported to National Drug Misuse Database held in the Drug Data Warehouse, DDW). These analyses from CPRD and DDW will be combined in a mathematical model of drug related mortality and drug treatment.

We will determine the risk of OD and death by treatment exposure and modality, relate these effects to the number of OD deaths and coverage and duration of drug treatment in the community, and project what factors could reduce the number of drug related deaths in the population.

Additional questions to be addressed include:-

- Is there a difference in the risk of mortality between prescribed methadone or buprenorphine?
- Is the risk of fatal overdose (OD) increased in people that are co-prescribed benzodiazepines?
- Is the risk of OD and mortality during and after residential, detoxification and non-pharmacological treatments similar to OST?
- Is the risk of OD and drug related death at treatment onset and cessation reduced in patients with evidence of supervised consumption and planned discharge?
- Is there evidence that delivery of OST in primary care has changed over time in terms of average dose, modality, duration, co-prescription of benzodiazepines, evidence of planned discharge and supervised consumption?
- Is there evidence that delivery of OST in primary care has changed over time – specifically in terms of changes in:- average dose and % of patients receiving optimal OST dose; OST modality; number and % of patients undergoing a planned discharged over time; number and % receiving co-prescription of benzodiazepines; number and % with evidence of supervised consumption.

**Study Type**

The study is observational and hypothesis testing (see aims/objectives above).

**Study Design**

We are conducting an observational “natural experiment” study and developing a policy impact model. The key intervention – Opiate Substitution Treatment (OST) can no longer be randomised with a placebo control, and no earlier trials of OST were large enough to assess mortality as the outcome(3). Therefore, we are exploiting information (that was not collected specifically for research) on variation in exposure and outcome derived from administrative databases. We will extract and analyse an open observational cohort based on Clinical Practice Research Datalink (CPRD) primary care database.

**Sample size/power calculation**

The main analysis is on opiate substation treatment (OST) in primary care and risk of death and in particular the comparison of methadone and buprenorphine*.

There will be at least 20,000 OST patients (extracted from CPRD GOLD patients) from 1998 to 2014 linked to NHS central registry and ONS mortality data, that will generate 67,000 person years (pys) follow-up (~32,000 on treatment and ~35,000 off) if censored 12 months after last OST. There will be over 700 deaths of which ~500 will be due to overdose. This will be the largest study in the UK with precise data on OST treatment exposure (including ~ 12000 pys on buprenorphine and 30,000pys on methadone). In addition, there will be approximately 10,000 and 12,000 person years in treatments lasting less than 1 month or greater than 12 months, and 3000 and 1200 person years follow-up in the first 4 weeks treatment on or immediately after methadone or buprenorphine treatment respectively.

Thus, there is sufficient power (> 90%) to determine whether:-

1. there is at least a halving of the risk of death during treatment on burpenorphine compared to methadone (i.e. 0.35% vs. 0.69% respectively) – which requires a sample of at least 922 in each group compared to 12,000 and 20,000 on buprenorophine and methadone
2. and sufficient power (80%) to test if the risk of death is at least a 1/3 lower on buprenorphine at (0.006%) compared to methadone (0.016%) in the first 4 weeks of treatment (1200 and 3000 person years respectively) – as indicated by unpublished analyses by Degenhardt on Australian mortality cohort(9).

In addition, there is sufficient power to corroborate that the risk of fatal overdose out of treatment at 0.8% is twice as high as the risk during treatment – which will require sample of at least 788 in and out of treatment compared to our sample of over 30,000 person years in each group; and that the risk of death in the 4 weeks after treatment at about 4.6% is 1/3 lower (3.0%) for people treated for short duration of treatment (< 1 month) compared to those treated for 12 months or longer – which will require sample of at least 3123 in each group compared to over 3500 and 3850 person years among those exposed to short or prolonged treatment respectively;

[In addition, the DDW linked mortality file has over 50,000 patients exposed to other structured non-pharmacological drug treatments, with around 44,000 pys of observation, from 2005 to 2009, and therefore also has sufficient power to test for differences in the risk of death in different periods during and after drug treatment.]

**Study population**

The study population comprise all patients aged 16-64 who have been prescribed methadone or buprenorphine as opiate substitution treatment (OST) in CPRD practices from 1997 to 2014, including a sub-set of patients eligible for ONS linkage. opiate dependent people treated with opiate substitution drugs on CPRD. The table shows the initial set of criteria. Prescription dose and diagnoses will be checked to confirm that prescription for OST and to exclude any patients prescribed for pain/analgesia.

| **prodcode** | **Productname** |
| --- | --- |
| 3064 | Buprenorphine 400microgram sublingual tablets sugar free |
| 3522 | Temgesic 200microgram sublingual tablets (RB Pharmaceuticals Ltd) |
| 6056 | Buprenorphine 8mg sublingual tablets sugar free |
| 6210 | Subutex 8mg sublingual tablets (RB Pharmaceuticals Ltd) |
| 6547 | Buprenorphine 2mg sublingual tablets sugar free |
| 7457 | Temgesic 0.3mg/ml Injection (Reckitt Benckiser Healthcare (UK) Ltd) |
| 8017 | Temgesic 400microgram sublingual tablets (RB Pharmaceuticals Ltd) |
| 10077 | Subutex 2mg sublingual tablets (RB Pharmaceuticals Ltd) |
| 13031 | Subutex 0.4mg sublingual tablets (RB Pharmaceuticals Ltd) |
| 35269 | Temgesic 300micrograms/1ml solution for injection ampoules (RB Pharmaceuticals Ltd) |
| 40211 | Buprenorphine 2mg sublingual tablets sugar free (Teva UK Ltd) |
| 40212 | Buprenorphine 8mg sublingual tablets sugar free (Teva UK Ltd) |
| 40473 | Buprenorphine 300micrograms/1ml solution for injection ampoules |
| 35169 | Suboxone 8mg/2mg sublingual tablets (RB Pharmaceuticals Ltd) |
| 35170 | Suboxone 2mg/500microgram sublingual tablets (RB Pharmaceuticals Ltd) |
| 35681 | Buprenorphine 2mg / Naloxone 500microgram sublingual tablets sugar free |
| 35682 | Buprenorphine 8mg / Naloxone 2mg sublingual tablets sugar free |
| 38311 | Naloxone hc 2mg + 8mg Tablet |
| 42074 | Naloxone hc 500micrograms + 2mg Tablet |
| 2952 | Methadone 1mg/ml oral solution |
| 5211 | Methadone 2mg/5ml linctus |
| 5322 | Physeptone 5mg tablets (Martindale Pharmaceuticals Ltd) |
| 6441 | Methadone 5mg tablets |
| 9728 | Methadone 1mg/ml oral solution sugar free |
| 11722 | Methadone 10mg/ml oral solution sugar free |
| 14086 | Methadone 10mg/ml Injection |
| 17671 | Methadone 50mg/1ml solution for injection ampoules |
| 17896 | Physeptone 10mg/ml Injection (Martindale Pharmaceuticals Ltd) |
| 21562 | Physeptone 1mg/ml mixture sugar free (Martindale Pharmaceuticals Ltd) |
| 23158 | Methadone 20mg/ml oral solution sugar free |
| 24343 | Methadose 10mg/ml oral solution concentrate (Rosemont Pharmaceuticals Ltd) |
| 24361 | Methadose 20mg/ml oral solution concentrate (Rosemont Pharmaceuticals Ltd) |
| 24440 | Methodex 1mg/ml Mixture (Link Pharmaceuticals Ltd) |
| 24584 | Methadone 50mg/2ml solution for injection ampoules |
| 26277 | Physeptone 1mg/ml mixture (Martindale Pharmaceuticals Ltd) |
| 28328 | Metharose 1mg/ml oral solution sugar free (Rosemont Pharmaceuticals Ltd) |
| 29304 | Physeptone 10mg/1ml solution for injection ampoules (Martindale Pharmaceuticals Ltd) |
| 29769 | Methadone 2mg/5ml Oral solution (Martindale Pharmaceuticals Ltd) |
| 29911 | Methadone 2mg/5ml linctus (Thornton & Ross Ltd) |
| 30531 | Methadone 1mg/ml oral solution sugar free (Rosemont Pharmaceuticals Ltd) |
| 32237 | Methex 1mg/ml Mixture (Generics (UK) Ltd) |
| 33068 | Methadone 10mg/1ml solution for injection ampoules |
| 33475 | Methadone 35mg/ml Injection |
| 33832 | Methadone 1mg/ml oral solution (Martindale Pharmaceuticals Ltd) |
| 35506 | Methadone 20mg/2ml solution for injection ampoules |
| 36436 | Methadone 50mg/5ml solution for injection ampoules |
| 36994 | Methadone 5mg/ml oral solution |
| 37507 | Physeptone 50mg/1ml solution for injection ampoules (Martindale Pharmaceuticals Ltd) |
| 37518 | Methadone 35mg/3.5ml solution for injection ampoules |
| 39437 | Eptadone 1mg/ml oral solution (Dee Pharmaceuticals Ltd) |
| 41608 | Methadone 1mg/ml oral solution (Rosemont Pharmaceuticals Ltd) |
| 41720 | Methadone 1mg/ml Mixture (Macarthy Medical Ltd) |
| 43260 | Methadone Oral solution |
| 43766 | Eptadone 5mg/ml oral solution (Dee Pharmaceuticals Ltd) |

**Selection of comparison group(s) or controls**

Patients are their own comparison group/ controls – as we will be comparing risk of mortality at different periods during and after opiate substitution treatment.

**Exposures, outcomes and covariates**

*Exposure:-* OST includes methadone and buprenorphine and occasionally dihydrocodeine(6, 11). Intervention exposure is defined by prescribing data collected by primary care practices contributing information through their electronic health record systems to the Clinical Practice Research Datalink (CPRD). This will include information on all prescriptions in primary care plus other information on diagnoses and attendances at primary care and hospital. We will extract information on patients on the basis that they have a drug misuse diagnosis and have been prescribed methadone or buprenorphine. Some patients also may have been prescribed dihydrocodeine as OST – and so we will include these treatment episodes as well. The information provided by CPRD will allow us to establish when OST started, changes in dose or type of OST, and when OST ceased. For each treatment period we will classify different periods “on”/during or “off”/after OST or other drug treatment e.g. 1st 2 weeks of treatment, rest of time on treatment (baseline treatment period), 1st 4 weeks after treatment, and rest of time out of treatment (censored at 12 months after last prescription); as well as measure average OST duration and classify treatment duration (≤ 1 month, 2-6 months, 7-12 months, 12+months).

*Main outcome:* All and cause specific death (in particular drug poisoning/overdose) during drug treatment or follow-up period up to one year after date of last opiate substitution treatment (OST) prescription/treatment admission. Standard codes for drug related poisonings deaths will be used to define overdose deaths (23): ICD-10 codes: F11-16; F18-19; X40-44; X60-64; X85; Y10-14; as well as in terms of general and specific drugs mentioned on the death certificate (any opiate, heroin and Morphine, Methadone, buprenorphine, dihydrocodeine, other opiods, cocaine, all amphetamines and MDMA, GHB, GBL, all benzodiazepines, barbiturates, all antidepressants, paracetamol and other over the counter medicines). We will categorise deaths as “avoidable” and “preventable” based on ONS definitions (<http://www.ons.gov.uk/ons/publications/re-reference-tables.html?edition=tcm%3A77-262418>). Other specific causes that will be investigated include suicide and other injury (non drug related poisoning), infectious diseases, cirrhosis and liver disease. All cause mortality will be expressed as a rate and as a Standardised Mortality Ratio (SMR) compared to standard age and sex death rates for England and Wales. Cause specific mortality will be expressed as rates – and SMRs also will be calculated for non-drug related poisonings. Follow-up of patients extracted from primary care database (Clinical Practice Research Datalink, CRPD) will be from date of first OST prescription within risk period beginning 1990 to end 2011. A patient’s follow up will cease either one year after the expiry of their last prescription for OST or the date of death or on the date of transfer out from the general practice or risk study period end (which ever date occurs first). [Follow-up of patients extracted from Drug Data Warehouse (DDW) will be from 2005 to 2009 the period for which National Drug Treatment records are linked to the mortality register.] We are censoring follow-up twelve months after last prescription or specialist drug treatment date in order to reduce potential bias.

Secondary outcomes concern the practice and delivery of OST including:- annual number of people in OST; modality/specific OST drug; duration of OST; dose; evidence of reaching optimal dosage, tapering OST dose towards planned discharge, and supervised consumption; and co-prescription of benzodiazepines.

*Covariates/confounders:-* Additional exposures (benzodiazepines, treatment performance/quality) and confounders (age, gender, co-morbidity as defined by Charlson Index(24), alcohol and mental health problems, treatment episode number, calendar year, other prescription drugs) all of which have been shown to influence mortality risk(11) (9). Supervised prescription will need to be coded through analysis of free-text – which will be piloted and developed with CPRD. A sample of records with full free text will be used to identify key words and search terms that can define the variable “evidence of supervised consumption”. The key words and search terms can then be used across the whole database to record the covariate.

**Use of linked data**

We will use link to ONS mortality file for our outcome measures. **Data/ Statistical Analysis Plan**

1) We expect that overdose and drug related mortality risk differs by period of treatment exposure (1-4 weeks treatment start, rest of treatment duration, 1-4 weeks treatment end, 5-52 weeks out of treatment). The main analysis will test whether there is evidence of a difference in mortality risk by modality of OST (methadone vs. buprenorphine) at different periods on and off treatment – and will investigate also mortality risk in relation to additional exposures (co-prescription of benzodiazepines, measures of treatment performance/quality) and confounders (age, gender, co-morbidity as defined by Charlson Index(24), alcohol and mental health problems, treatment episode number, calendar year) all of which have been shown to influence mortality risk(11) (9). However, other potential confounders or factors relating to injecting risk may be missing or incompletely measured in CRPD (such as crack-cocaine use).

We will undertake a series of analyses that aim to incorporate and adjust for confounding.

First, we will use Poisson regression incorporating time varying covariates (such as co-prescription of benzodiazepines, episode and duration of treatment) to describe mortality risks and compare these at different periods on and off treatment and by modality of OST. These analyses will generate incidence rate ratios (IRR) that compare mortality risk for patients on methadone (from week 4 to end of treatment as baseline) to other periods and other forms of OST; will compare mortality rate for key confounders (above); adjust IRR by period on or off treatment for these confounders; and examine specifically whether co-prescription of benzodiazpeines increases the risk of mortality during and after treatment.

Second, we will undertake analyses of patient characteristics in order to develop and use propensity scores that can be used to balance buprenorphine and methadone exposure groups and test for differences in mortality risk between the groups(25-27). The primary care databases hold a substantial amount of information on the patients’ diagnoses and treatment history as well as information on the practices – too much data to fit as individual covariates. Therefore, we can use these data to generate propensity score matching in order to use the score to reduce selection bias and match the patients exposed either to buprenorphine or methadone or to short or long term treatment(27).

Third, the problem of confounding (known and unknown) also will be addressed using self-controlled case-series (SCCS) methodology in which patients act as their own controls(28), as has been used in other studies of primary care databases(29, 30). In this method the number of events are classified according to the length of the exposure (in period; in and out of treatment) and an estimate of the hazard or incidence rate ratio is derived by conditioning on the number of events over the exposure history(28). Since there is missing information on risk of death prior to exposure only cases that have been exposed are included in the analysis. In addition, time invariant confounders (such as sex, age, underlying health problems) are controlled for implicitly; and time variant confounders can be fitted within the model. Prof Farrington and Dr Whitaker are extending the SCCS method to deal with finite outcomes such as drug related/overdose death, and exposures that may persist (such as opiate substitution treatment) and this study will be a case study on how SCCS can be used with such exposures and outcomes. SCCS has been used for censored events – such as death previously (31, 32) – but classifying the exposure will require methodological development.

Fourth, even in the absence of unmeasured confounding, conventional methods (eg regression models that control for baseline or time-dependent confounders) will provide estimates that may fail to have a causal interpretation when (i) there is a measured time-dependent risk factor for survival that also predicts subsequent treatment, and (ii) past treatment history predicts the subsequent level of this risk factor. We will use inverse probability (IP) weighted estimation of marginal structural models, which avoids conditioning on time-dependent confounders(33, 34). Instead, we estimate the inverse of each patient’s probability of their observed treatment history at each time. By weighting a marginal model according to the reciprocal of these probabilities, we mimic a situation in which the assignment to treatment is at random (or unrelated to the time-varying covariates).

By examining the intervention effects between exposure and outcome and how they vary across multiple methods which adjust for confounding in different ways we will be able to assess the likelihood that residual/time-varying confounding remains a significant problem, and to contribute stronger evidence on the impact of treatment on drug related mortality(35, 36). Similar analyses will be conducted on the risk of mortality during and after exposure to non-pharmacological treatments(18).

2) The secondary analysis of clinical databases will test whether there have been changes over time in the number of patients treated over time and specifically in the delivery of OST in primary care (e.g. the mean dose, treatment duration, modality and evidence of supervised consumption). Segmented regression techniques will be used for time trend analyses of these treatment indicators in order to test whether there have been changes over time, and whether the timing of the change is consistent with issuing of new treatment guidelines(20, 37).

3) The estimates of the effect of different drug treatments on mortality risk for different periods on or off treatment derived from analyses of Clinical Practice Research Datalink (CPRD) and Drug Data Warehouse (DDW) will be used in differential equation or individual based models that consider the flow of people into and out of drug treatment fitted against estimates of the annual number of drug related deaths in the population. The models will incorporate treatment coverage and extend previous models(11) by including additional factors that may affect the risk of overdose death or duration maintained on treatment in this analysis. This will be decided through consultation with other researchers on the grant and key stakeholders (Public Health England, Bristol Health Integration Team on Addiction) and may include:- modality of treatment, combination of treatments, age, gender, treatment history, benzodiazepine prescription, alcohol abuse, other psychosocial and family therapy (if data on intervention effect are available). An individual based model will be used if it is deemed important to incorporate the full heterogeneity in treatment duration and/or if an individual’s past treatment episodes are shown to effect the duration that they remain on treatment or the mortality rate while on treatment. The model will be parameterized with data from this study, and key uncertainties will be varied in a Bayesian framework to obtain multiple possible model fits to the annual number of drug related deaths in the population at certain times points in the past(38). Model fits will be weighted by their goodness of fit using likelihood measures (39, 39).

The model fits for each time point will be used to firstly evaluate the impact of current and past levels of drug treatment on decreasing drug related deaths in the population (11, 22), with model fits being used to test hypotheses for why the yearly number of overdose deaths has not decreased over the last 10 years while the rate of methadone deaths per gram has decreased. The hypotheses will be incorporated into the model fitting process through assumptions made about the uncertainty ranges or values assumed for specific parameters, and then the likelihood of specific hypotheses will be evaluated through the relative degree to which the model fits the data with that parameter assumption(40, 41).

More importantly the models will then be used to project the likely impact of varying specific current characteristics of drug treatment on the incidence of drug related deaths in the population, such as: combining different treatment modalities, changing treatment duration, controlling concomitant prescription, and introducing interventions to reduce treatment drop-out or relapse following treatment cessation(11, 22). These projections will increase our understanding of the role of different factors in effecting the impact of OST, and the possible impact of future strategies that may attempt to reduce overdose death by altering certain characteristics of drug treatment in the UK. The relative efficiency of these strategies will also be assessed with efficiency being defined in terms of the number of deaths averted per person year on OST. This will help us to assess the likely optimum strategies for reducing the frequency of overdose deaths for specific coverage levels of drug treatment in terms of grams distributed per year.

**Patient/ user group involvement**

Members of M-Alliance will be on our project advisory group and contribute to the interpretation, reporting and dissemination of the research. The M-Alliance is a user led organization which provides advocacy, training and helpline services to those currently in drug or alcohol treatment, those who have accessed treatment in the past and those who may access treatment in the future (http://www.m-alliance.org.uk/index.html). The M-Alliance has an ongoing interest in improving access to effective drug treatment, especially opiate substitution treatment (OST), for those that would benefit from treatment. Key policy statements recently by the M-Alliance have raised concern over the potential impact of shortening the duration of treatment (such as http://www.ukhra.org/putting_public_health_first/); and highlighted the importance of strengthening the evidence base on the benefits of treatment. Our previous analyses of the risk of drug related death during and after OST(11) were of interest and discussed with the M-Alliance who were keen to see these analyses extended and repeated. In particular there is a common interest in assessing the comparative effectiveness of methadone and buprenorphine – and sharing the findings with other users and drug workers. Financial support for members of the Alliance to participate in project meetings will be provided through the project grant. It is unlikely that M-Alliance participants will require training as we will be benefiting from their expertise, however, we will consult with NIHR PPI leads to provide advice at the beginning of the study. The Alliance will support research dissemination through summaries provided to their user fora, and through presentation to users and clinical practitioners at conferences such as the UK Harm Reduction Alliance conference on injecting.

**Limitations of the study design, data sources and analytic methods**

The key limitation is confounding – which we discuss and seek to address in our analysis plan above. Multiple analyses will be undertaken running the risk of “statistical multiplicity” and we will guard against over-interpretation of single statistical tests(42). The key hypothesis to be tested is whether there is evidence that buprenorphine is a safer substitution drug (in relation to mortality risk) than methadone. In addition, we will corroborate earlier analyses that hypothesised that mortality risk was elevated compared to other periods immediately after treatment cessation. Several analytic approaches will be presented in order to consider the extent of confounding and to aid interpretation of the analyses.

There are missing prescription data which may lead to misclassification of periods on and off (e.g. missing amount prescribed or daily dose in order to determine last date of prescription) – which we will subject to sensitivity analysis – including using different definitions/criteria for when a patient is on/off OST and using only the last prescription. Some patients maybe treated in specialist drug treatment clinics as well as primary care, which may introduce a potential bias if the information on other periods on OST is unrecorded. We will undertake analyses to estimate the frequency of this occurrence and how CPRD can be used to identify these periods. Periods in prison are unlikely to be recorded which also may introduce bias and confer additional risks (e.g. the period in prison will be at lower mortality risk but the period immediately following prison release is a period of high risk(43)). We can estimate the likely frequency and proportion of these events from other analyses to assess potential bias, and take these biases into account in the interpretation of our findings.

The analysis of CPRD will describe mortality risk for different periods on and off treatment for further mathematical models – which include other information – such as proportion of opiate dependent people in treatment. These also are subject to uncertainty and will be explored in sensitivity analyses.

**Plans for disseminating and communicating study results**

The study and findings will be discussed with key stakeholders at Public Health England (National Treatment Agency (NTA)), Department of Health, and Home Office (Drug and Alcohol Policy) with an interest in drug treatment and policy. We have presented earlier work on drug related mortality to NTA, and Advisory Council on Misuse of Drugs (ACMD), European Monitoring Centre on Drugs and Drug Addiction (EMCDDA), and meetings and conferences organised by US National Institute of Drugs and Addiction (NIDA). We will present the new information at invited meetings at PHE/NTA, ACMD and EMCDDA. In addition, we will also present the data to Bristol drug strategy team and local health partners which are developing a Health Integration Team (HIT) on Addiction in order better to support commissioning of local services. We will develop a relevant summary report and overheads that can be offered to other local agencies (public health in local government, CCGs and Health and Wellbeing Boards).

The research output will be presented in peer review publications and presented at national and international scientific meetings.

We will produce a lay summary of the findings and pod-cast developed in conjunction with M-Alliance and Exchange supplies. The summary and pod-cast will be distributed to peers and user networks.

Reference List

1. Ward J, Hall W, Mattick RP. Role of maintenance treatment in opioid dependence. Lancet 1999;353(9148):221-226.

2. Gowing L, Farrell MF, Bornemann R, Sullivan LE, Ali R. Oral substitution treatment of injecting opioid users for prevention of HIV infection. Cochrane Database Syst Rev 2011;(8):CD004145.

3. Mattick RP, Breen C, Kimber J, Davoli M. Methadone maintenance therapy versus no opioid replacement therapy for opioid dependence. Cochrane Database Syst Rev 2009;(3):CD002209.

4. Turner KM, Hutchinson S, Vickerman P, Hope V, Craine N, Palmateer N, et al. The impact of needle and syringe provision and opiate substitution therapy on the incidence of hepatitis C virus in injecting drug users: pooling of UK evidence. Addiction 2011 Nov;106(11):1978-1988.

5. Amato L, Davoli M, Perucci CA, Ferri M, Faggiano F, Mattick RP. An overview of systematic reviews of the effectiveness of opiate maintenance therapies: available evidence to inform clinical practice and research. Journal of Substance Abuse Treatment 2005;28(4):321-329.

6. Robertson JR, Raab GM, Bruce M, McKenzie JS, Storkey HR, Salter A. Addressing the efficacy of dihydrocodeine versus methadone as an alternative maintenance treatment for opiate dependence: A randomized controlled trial. Addiction 2006 Dec;101(12):1752-1759.

7. Godfrey C, Stewart D, Gossop M. Economic analysis of costs and consequences of the treatment of drug misuse: 2-year outcome data from the National Treatment Outcome Research Study (NTORS). Addiction 2004 Jun;99(6):697-707.

8. Caplehorn JRM, Dalton MSYN, Haldar F, Petrenas AM, Nisbet JG. Methadone maintenance and addicts' risk of fatal heroin overdose. Substance Use & Misuse 1996;31(2):177-196.

9. Degenhardt L, Randall D, Hall W, Law M, Butler T, Burns L. Mortality among clients of a state-wide opioid pharmacotherapy program over 20 years: risk factors and lives saved. Drug Alcohol Depend 2009 Nov 1;105(1-2):9-15.

10. Davoli M, Bargagli AM, Perucci CA, Schifano P, Belleudi V, Hickman M, et al. Risk of fatal overdose during and after specialist drug treatment: the VEdeTTE study, a national multi-site prospective cohort study. Addiction 2007 Dec;102(12):1954-1959.

11. Cornish R, Macleod J, Strang J, Vickerman P, Hickman M. Risk of death during and after opiate substitution treatment in primary care: prospective observational study in UK general practice research database. BMJ 2010;341:c5475.

12. Degenhardt L, Bucello C, Mathers B, Briegleb C, Ali H, Hickman M, et al. Mortality among regular or dependent users of heroin and other opioids: A systematic review and meta-analysis of cohort studies. Addiction 2010;106:32-51.

13. Mattick RP, Kimber J, Breen C, Davoli M. Buprenorphine maintenance versus placebo or methadone maintenance for opioid dependence. Cochrane Database Syst Rev 2008;(2):CD002207.

14. White JM, Irvine RJ. Mechanisms of fatal opioid overdose. Addiction 1999;94(7):961-972.

15. Auriacombe M, Fatseas M, Dubernet J, Daulouede JP, Tignol J. French field experience with buprenorphine. American Journal on Addictions 2004;13:S17-S28.

16. Buster MC, van Brussel GH, van den Brink W. An increase in overdose mortality during the first 2 weeks after entering or re-entering methadone treatment in Amsterdam. Addiction 2002 Aug;97(8):993-1001.

17. McCowan C, Kidd B, Fahey T. Factors associated with mortality in Scottish patients receiving methadone in primary care: retrospective cohort study. BMJ 2009;338(doi: 10.1136/bmj.b2225.).

18. Strang J, McCambridge J, Best D, Beswick T, Bearn J, Rees S, et al. Loss of tolerance and overdose mortality after inpatient opiate detoxification: follow up study. BMJ 2003 May 3;326(7396):959-960.

19. Morgan O, Griffiths C, Hickman M. Association between availability of heroin and methadone and fatal poisoning in England and Wales 1993-2004. Int J Epidemiol 2006 Dec;35(6):1579-1585.

20. Strang J, Hall W, Hickman M, Bird SM. The impact of supervised methadone consumption on opiate overdose deaths in England and Scotland: analysis using the OD4 Index. BMJ 2010;341:c4851.

21. Morgan O, Vicente J, Griffiths P, Hickman M. Trends in overdose deaths from drug misuse in Europe: what do the data tell us? Addiction 2008 May;103(5):699-700.

22. Hickman M, Vickerman P, Robertson R, Macleod J, Strang J. Promoting recovery and preventing drug-related mortality: competing risks? J Public Health (Oxf) 2011 Sep;33(3):332-334.

23. Morgan O, Griffiths C, Toson B, Rooney C, Majeed A, Hickman M. Trends in deaths related to drug misuse in England and Wales, 1993-2004. Health Stat Q 2006;(31):23-27.

24. Khan NF, Perera R, Harper S, Rose PW. Adaptation and validation of the Charlson Index for Read/OXMIS coded databases. BMC Fam Pract 2010;11:1.

25. Tannen RL, Weiner MG, Xie D. Use of primary care electronic medical record database in drug efficacy research on cardiovascular outcomes: comparison of database and randomised controlled trial findings. BMJ 2009;338:b81.

26. Rosenbaum P, Rubin D. The central role of the propensity score in observational studies for causal effects. Biometrika 1983;70:41-55.

27. D'Agostino RB, Jr. Propensity score methods for bias reduction in the comparison of a treatment to a non-randomized control group. Stat Med 1998 Oct 15;17(19):2265-2281.

28. Whitaker HJ, Farrington CP, Spiessens B, Musonda P. Tutorial in biostatistics: the self-controlled case series method. Stat Med 2006 May 30;25(10):1768-1797.

29. Hubbard R, Lewis S, West J, Smith C, Godfrey C, Smeeth L, et al. Bupropion and the risk of sudden death: a self-controlled case-series analysis using The Health Improvement Network. Thorax 2005 Oct;60(10):848-850.

30. Petersen I, Gilbert R, Evans S, Ridolfi A, Nazareth I. Oral antibiotic prescribing during pregnancy in primary care: UK population-based study. J Antimicrob Chemother 2010 Oct;65(10):2238-2246.

31. Kuhnert R, Hecker H, Poethko-Muller C, Schlaud M, Vennemann M, Whitaker HJ, et al. A modified self-controlled case series method to examine association between multidose vaccinations and death. Stat Med 2011 Mar 15;30(6):666-677.

32. Whitaker HJ, Hocine MN, Farrington CP. The methodology of self-controlled case series studies. Stat Methods Med Res 2009 Feb;18(1):7-26.

33. Robins JM, Hernan MA, Brumback B. Marginal structural models and causal inference in epidemiology. Epidemiology 2000 Sep;11(5):550-560.

34. Hernan MA, Brumback B, Robins JM. Marginal structural models to estimate the joint causal effect of nonrandomised treatments. Journal of American Statistical Association 2001;96:440-448.

35. MRC. Using natural experiments to evaluate population health interventions. London: MRC; 2011 Jul 11.

36. Stukel TA, Fisher ES, Wennberg DE, Alter DA, Gottlieb DJ, Vermeulen MJ. Analysis of observational studies in the presence of treatment selection bias: effects of invasive cardiac management on AMI survival using propensity score and instrumental variable methods. JAMA 2007 Jan 17;297(3):278-285.

37. Department of Health (England) and the devolved administrations. Drug Misuse and Dependence: UK Guidelines on Clinical Management. London: Department of Health; 2007.

38. Vickerman P, Foss AM, Pickles M, Deering K, Verma S, Eric D, et al. To what extent is the HIV epidemic in southern India driven by commercial sex? A modelling analysis. Aids 2010 Oct 23;24(16):2563-2572.

39. Hallett TB, Gregson S, Mugurungi O, Gonese E, Garnett GP. Assessing evidence for behaviour change affecting the course of HIV epidemics: a new mathematical modelling approach and application to data from Zimbabwe. Epidemics 2009 Jun;1(2):108-117.

40. Boily MC, Pickles M, Lowndes CM, Ramesh BM, Washington R, Moses S, et al. Positive impact of a large-scale HIV prevention program among female sex workers and clients in Karnataka state, India. Aids 2013 Mar 3.

41. Vickerman P, Platt L, Hawkes S. Modelling the transmission of HIV and HCV among injecting drug users in Rawalpindi, a low HCV prevalence setting in Pakistan. Sex Transm Infect 2009 Apr;85 Suppl 2:ii23-ii30.

42. Sterne JA, Davey SG. Sifting the evidence-what's wrong with significance tests? BMJ 2001 Jan 27;322(7280):226-231.

43. Farrell M, Marsden J. Acute risk of drug-related death among newly released prisoners in England and Wales. Addiction 2008 Feb;103(2):251-255.

ADMENDMENT 3^rd^ November 2014

**Study population**

Data extraction comprises all patients aged 16-64 who have been prescribed methadone, buprenorphine or dihydrocodeine in CPRD practices from 1997 to 2014. Patients solely prescribed dihydrocodeine will require additional evidence of drug addiction. This will include a sub-set of patients eligible for ONS linkage.

Patients prescribed methadone or buprenorphine will be identified from the following product codes.

Additional patients will be identified based upon prescriptions for dihydrocodeine using the following list of product codes

and evidence of drug addiction from the following list of medcodes.

The study population will be a subset of the data extraction based upon prescription dose and diagnoses to confirm that prescriptions were for opiate substitution treatment (OST) and to exclude any patients prescribed for pain/analgesia.

AMENDMENT 9^th^ December 2015

**Use of linked data**

We will use the ONS mortality file to define overdose and drug related mortality. Previously supplied data in 2014 provided fewer deaths than expected with only 30% of deaths in the OST sample being identified as overdose or drug related. This left analyses underpowered to detect expected effect sizes. The new linkage data, set 11 covering a greater number of practices and a longer time span, offers the potential to overcome the limitations of our current data set.
