# Supplementary material for: Prescription of benzodiazepines, z-drugs, and gabapentinoids and mortality risk in people receiving opioid agonist treatment: Observational study based on the UK Clinical Practice Research Datalink and Office for National Statistics death records
Source: PLoS Med. 2019 Nov 26;16(11):e1002965. doi: 10.1371/journal.pmed.1002965 (PMC6879111; doi:10.1371/journal.pmed.1002965)
Supplement: S1 STROBE Checklist — (DOCX) [file pmed.1002965.s004.docx]

STROBE Statement—Checklist of items that should be included in reports of ***cohort studies***

|  | Item No | Recommendation |
| --- | --- | --- |
| **Title and abstract** | 1 | (*a*) Indicate the study’s design with a commonly used term in the title or the abstract Observational study based on the UK Clinical Practice Research Datalink and Office of National Statistics death records |
|  |  | (*b*) Provide in the abstract an informative and balanced summary of what was done and what was found  **STRUCTURED ABSTRACT PAGES 2-3** |
| Introduction | | |
| Background/rationale | 2 | Explain the scientific background and rationale for the investigation being reported  **INTRODUCTION PAGES 6-7** |
| Objectives | 3 | State specific objectives, including any prespecified hypotheses  **INTRODUCTION FINAL PARAGRAPH.** |
| Methods | | |
| Study design | 4 | Present key elements of study design early in the paper  **STRUCTURED ABSTRACT, INTRODUCTION FINAL PARAGRAPH** |
| Setting | 5 | Describe the setting, locations, and relevant dates, including periods of recruitment, exposure, follow-up, and data collection  **METHODS PARAGRAPHS 3-5** |
| Participants | 6 | (*a*) Give the eligibility criteria, and the sources and methods of selection of participants. Describe methods of follow-up. **METHODS PARAGRAPHS 3-5** |
|  |  | (*b*) For matched studies, give matching criteria and number of exposed and unexposed. **NO MATCHING BUT PARTICIPANT CHARACTERISTICS DESCRIBED IN TABLE 1.** |
| Variables | 7 | Clearly define all outcomes, exposures, predictors, potential confounders, and effect modifiers. Give diagnostic criteria, if applicable **METHODS PARAGRAPHS 3-6** |
| Data sources/ measurement | 8* | For each variable of interest, give sources of data and details of methods of assessment (measurement). Describe comparability of assessment methods if there is more than one group **METHODS PARAGRAPHS 3-6** |
| Bias | 9 | Describe any efforts to address potential sources of bias **METHODS PARAGRAPHS 5-10** |
| Study size | 10 | Explain how the study size was arrived at **DESCRIBED IN METHODS AND IN** **WEB FIGURE 2** |
| Quantitative variables | 11 | Explain how quantitative variables were handled in the analyses. If applicable, describe which groupings were chosen and why **METHODS PARAGRAPHS 3-10** |
| Statistical methods | 12 | (*a*) Describe all statistical methods, including those used to control for confounding **METHODS PARAGRAPHS 7-9** |
|  |  | (*b*) Describe any methods used to examine subgroups and interactions  **METHODS PARAGRAPH 8** |
|  |  | (*c*) Explain how missing data were addressed **NOT APPLICABLE** |
|  |  | (*d*) If applicable, explain how loss to follow-up was addressed **NO LOSS TO FOLLOW-UP BUT CENSORING EXPLAINED** |
|  |  | (*e*) Describe any sensitivity analyses **METHODS PARAGRAPH 9** |
| Results | | |
| Participants | 13* | (a) Report numbers of individuals at each stage of study—eg numbers potentially eligible, examined for eligibility, confirmed eligible, included in the study, completing follow-up, and analysed **WEB FIGURE 2** |
|  |  | (b) Give reasons for non-participation at each stage **N/A** |
|  |  | (c) Consider use of a flow diagram **WEB FIGURE 2** |
| Descriptive data | 14* | (a) Give characteristics of study participants (eg demographic, clinical, social) and information on exposures and potential confounders **TABLE 1** |
|  |  | (b) Indicate number of participants with missing data for each variable of interest **N/A** |
|  |  | (c) Summarise follow-up time (eg, average and total amount) **RESULTS PARAGRAPH 1** |
| Outcome data | 15* | Report numbers of outcome events or summary measures over time**TABLE 4** |
| Main results | 16 | (*a*) Give unadjusted estimates and, if applicable, confounder-adjusted estimates and their precision (eg, 95% confidence interval). Make clear which confounders were adjusted for and why they were included **TABLES 2 - 6** |
|  |  | (*b*) Report category boundaries when continuous variables were categorized **N/A** |
|  |  | (*c*) If relevant, consider translating estimates of relative risk into absolute risk for a meaningful time period **N/A** |
| Other analyses | 17 | Report other analyses done—eg analyses of subgroups and interactions, and sensitivity analyses **METHODS AND APPENDIX** |
| Discussion | | |
| Key results | 18 | Summarise key results with reference to study objectives –**DISCUSSION PARAGRAPHS 1-3** |
| Limitations | 19 | Discuss limitations of the study, taking into account sources of potential bias or imprecision. Discuss both direction and magnitude of any potential bias **DISCUSSION PARAGRAPH 7** |
| Interpretation | 20 | Give a cautious overall interpretation of results considering objectives, limitations, multiplicity of analyses, results from similar studies, and other relevant evidence **DISCUSSION PARAGRAPHS 1-8** |
| Generalisability | 21 | Discuss the generalisability (external validity) of the study results **DISCUSSION PARAGRAPH 7** |
| Other information | | |
| Funding | 22 | Give the source of funding and the role of the funders for the present study and, if applicable, for the original study on which the present article is based **GIVEN** |

*Give information separately for exposed and unexposed groups.

**Note:** An Explanation and Elaboration article discusses each checklist item and gives methodological background and published examples of transparent reporting. The STROBE checklist is best used in conjunction with this article (freely available on the Web sites of PLoS Medicine at http://www.plosmedicine.org/, Annals of Internal Medicine at http://www.annals.org/, and Epidemiology at http://www.epidem.com/). Information on the STROBE Initiative is available at http://www.strobe-statement.org.
