# Supplementary material for: Prescription of benzodiazepines, z-drugs, and gabapentinoids and mortality risk in people receiving opioid agonist treatment: Observational study based on the UK Clinical Practice Research Datalink and Office for National Statistics death records
Source: PLoS Med. 2019 Nov 26;16(11):e1002965. doi: 10.1371/journal.pmed.1002965 (PMC6879111; doi:10.1371/journal.pmed.1002965)
Supplement: S1 Table — (DOCX) [file pmed.1002965.s005.docx]

**Definition of drug related deaths**

| **Description** | **ICD-9 Codes** | **ICD-10 Codes** |
| --- | --- | --- |
| Mental and behavioural disorders |  |  |
| due to drug use (excluding alcohol and tobacco) ^a^ | 292, 304, 305.2–305.9 | F11–F16, F18–F19 |
| Unspecified cause/disorder |  | F99 |
| Accidental self-harm |  |  |
| Poisoning by drugs, medicaments and biological substances ^a^ | E850–E858 | X40–X44 |
| Poisoning, other or unspecified exposure | E866.8, E866.9 | X49 |
| Other or unspecified means | E928.8, E928.9 | X58, X59.9 |
| Intentional self-harm |  |  |
| Poisoning by drugs, medicaments and biological substances ^a^ | E950.0–E950.5 | X60–X64 |
| Poisoning, other or unspecified exposure | E950.9 | X69 |
| Other or unspecified means | E958.8, E958.9 | X83, X84 |
| Assault by |  |  |
| Poisoning by drugs, medicaments and biological substances ^a^ | E962.0 | X85 |
| Poisoning, other or unspecified exposure | E962.9 | X90 |
| Other or unspecified means | E968.8, E968.9 | Y08, Y09 |
| Self-harm, undetermined intent |  |  |
| Poisoning by drugs, medicaments and biological substances ^a^ | E980.0–E980.5 | Y10–Y14 |
| Poisoning, other or unspecified exposure | E980.9 | Y19 |
| Other or unspecified means | E988.8, E988.9 | Y33, Y34 |
| External cause |  |  |
| Poisoning by drugs, medicaments and biological substances | 960-979 | T36-T50 |
| Poisoning, other or unspecified exposure | 989.89, 989.9 | T65.8, T65.9 |
| Other or unspecified cause | 995.89 | T78.8, T78.9 |
|  |  |  |
| Ill-defined, unpecified or unknown cause | 798.1–798.9, 799.89, 799.9 | R68.8, R69, R96-R99 |

^a^ ONS (2015), Statistical Bulletin: Deaths Related to Drug Poisoning in England and Wales 2014, p33
