# Supplementary material for: Prescription of benzodiazepines, z-drugs, and gabapentinoids and mortality risk in people receiving opioid agonist treatment: Observational study based on the UK Clinical Practice Research Datalink and Office for National Statistics death records
Source: PLoS Med. 2019 Nov 26;16(11):e1002965. doi: 10.1371/journal.pmed.1002965 (PMC6879111; doi:10.1371/journal.pmed.1002965)
Supplement: S2 Table — (DOCX) [file pmed.1002965.s006.docx]

**Benzodiazepine and z-drug dose criteria**

|  | High criterion ^a^ | Number | % high |
| --- | --- | --- | --- |
| Drug | (mg) | Prescriptions | (> criterion) |
| **Benzodiazepine** |  |  |  |
| alprazolam | 1.5 | 133 | 33.08 |
| chlordiazepoxide | 100 | 4633 | 10.55 |
| diazepam | 30 | 237914 | 21.35 |
| flurazepam | 30 | 21 | 100.00 |
| loprazolam | 2 | 440 | 30.23 |
| lorazepam | 4 | 2540 | 8.90 |
| lormetazepam | 1.5 | 978 | 36.40 |
| nitrazepam | 10 | 52982 | 35.13 |
| oxazepam | 120 | 460 | 0.00 |
| temazepam | 40 | 65481 | 10.80 |
| **Z-drug** |  |  |  |
| zaleplon | 10 | 182 | 3.85 |
| zolpidem | 10 | 6079 | 11.35 |
| zopiclone | 7.5 | 69665 | 52.21 |
| Total |  | 441508 | 26.01 |

^a^ Maximum recommended daily dose as defined in BNF
