# Supplementary material for: Prescription of benzodiazepines, z-drugs, and gabapentinoids and mortality risk in people receiving opioid agonist treatment: Observational study based on the UK Clinical Practice Research Datalink and Office for National Statistics death records
Source: PLoS Med. 2019 Nov 26;16(11):e1002965. doi: 10.1371/journal.pmed.1002965 (PMC6879111; doi:10.1371/journal.pmed.1002965)
Supplement: S3 Table — (DOCX) [file pmed.1002965.s007.docx]

**All cause, drug related and non-drug related mortality rates for covariates included in the analyses**

| Confounder | | All cause mortality | | | | Drug related poisoning | | | | Non-drug related mortality | | | |
| --- | --- | --- | --- | --- | --- | --- | --- | --- | --- | --- | --- | --- | --- |
|  |  | Deaths | PY | MR | P | Deaths | PY | MR | p | Deaths | PY | MR | P |
| Gender | Male | 428 | 24904 | 1.72 | 0.0359 | 99 | 13807 | 0.72 | 0.0001 | 175 | 14087 | 1.24 | 0.0051 |
|  | Female | 229 | 11222 | 2.04 |  | 14 | 6142 | 0.23 |  | 110 | 6299 | 1.75 |  |
| Age | <30 | 58 | 7319 | 0.79 | <0.0001 | 27 | 4131 | 0.65 | 0.6068 | 13 | 4047 | 0.32 | <0.0001 |
|  | 30-39 | 136 | 14918 | 0.91 |  | 49 | 8082 | 0.61 |  | 33 | 8080 | 0.41 |  |
|  | 40-49 | 166 | 8930 | 1.86 |  | 24 | 4959 | 0.48 |  | 62 | 5072 | 1.22 |  |
|  | 50+ | 297 | 4959 | 5.99 |  | 13 | 2776 | 0.47 |  | 177 | 3186 | 5.55 |  |
| Year | 1998-99 | 45 | 1799 | 2.50 | 0.1055 | 14 | 928 | 1.51 | 0.0007 | 14 | 978 | 1.43 | 0.3390 |
|  | 2000-04 | 151 | 9025 | 1.67 |  | 31 | 4592 | 0.68 |  | 53 | 4724 | 1.12 |  |
|  | 2005-09 | 245 | 13868 | 1.77 |  | 33 | 7793 | 0.42 |  | 119 | 7959 | 1.50 |  |
|  | 2010-14 | 216 | 11434 | 1.89 |  | 35 | 6634 | 0.53 |  | 99 | 6725 | 1.47 |  |
| Comorbidity | 0 | 222 | 25225 | 0.88 | <0.0001 | 68 | 13901 | 0.49 | 0.0041 | 68 | 13970 | 0.49 | <0.0001 |
| Score | 1 | 110 | 8632 | 1.27 |  | 31 | 4959 | 0.63 |  | 38 | 5099 | 0.75 |  |
|  | 2+ | 325 | 2269 | 14.32 |  | 14 | 1088 | 1.29 |  | 179 | 1317 | 13.60 |  |
| Region | North East | 10 | 498 | 2.01 | <0.0001 | 0 | 455 | 0.00 | 0.1749 | 8 | 479 | 1.67 | <0.0001 |
|  | North West | 75 | 5458 | 1.37 |  | 33 | 4432 | 0.74 |  | 35 | 4448 | 0.79 |  |
|  | Yorkshire/Humber | 33 | 1567 | 2.11 |  | 3 | 911 | 0.33 |  | 16 | 942 | 1.70 |  |
|  | East Midlands | 32 | 1476 | 2.17 |  | 4 | 744 | 0.54 |  | 13 | 747 | 1.74 |  |
|  | West Midlands | 53 | 4059 | 1.31 |  | 14 | 3297 | 0.42 |  | 31 | 3350 | 0.93 |  |
|  | East | 93 | 2730 | 3.41 |  | 16 | 1827 | 0.88 |  | 66 | 1896 | 3.48 |  |
|  | South West | 49 | 3160 | 1.55 |  | 17 | 2921 | 0.58 |  | 31 | 2990 | 1.04 |  |
|  | South Central | 46 | 3486 | 1.32 |  | 8 | 2506 | 0.32 |  | 23 | 2557 | 0.90 |  |
|  | London | 65 | 2607 | 2.49 |  | 9 | 1954 | 0.46 |  | 47 | 2070 | 2.27 |  |
|  | South East | 28 | 1134 | 2.47 |  | 9 | 901 | 1.00 |  | 15 | 906 | 1.66 |  |
|  | Northern Ireland | 2 | 174 | 1.15 |  |  |  |  |  |  |  |  |  |
|  | Scotland | 132 | 7429 | 1.78 |  |  |  |  |  |  |  |  |  |
|  | Wales | 39 | 2347 | 1.66 |  |  |  |  |  |  |  |  |  |

*PY – person years follow-up; MR mortality rate (deaths/100 person-years). P (Unadjusted P values testing for differences in mortality rates by confounders).*
