# Supplementary material for: Prescription of benzodiazepines, z-drugs, and gabapentinoids and mortality risk in people receiving opioid agonist treatment: Observational study based on the UK Clinical Practice Research Datalink and Office for National Statistics death records
Source: PLoS Med. 2019 Nov 26;16(11):e1002965. doi: 10.1371/journal.pmed.1002965 (PMC6879111; doi:10.1371/journal.pmed.1002965)
Supplement: S4 Table — (DOCX) [file pmed.1002965.s008.docx]

**Poisson regression analyses showing incident rate ratios for ACM, DRP and non-DRP according to co-prescribed medications**

|  | Unadjusted | | Adjusted* | |
| --- | --- | --- | --- | --- |
| Exposure | IRR (95% CI) | P | IRR (95% CI) | P |
| **All cause** |  |  |  |  |
| B Off | 1 (ref) | 0.3263 | 1 (ref) | 0.0907 |
| B On | 1.10 (0.91 to 1.32) |  | 1.18 (0.97 to 1.42) |  |
| B Off | 1 (ref) | 0.6154 | 1 (ref) | 0.2025 |
| B On normal | 1.10 (0.90 to 1.35) |  | 1.21 (0.98 to 1.48) |  |
| B On high | 1.08 (0.74 to 1.58) |  | 1.07 (0.72 to 1.58) |  |
| B Linear | 1.07 (0.92 to 1.23) | 0.3738 | 1.11 (0.96 to 1.28) | 0.1681 |
| Z Off | 1 (ref) | 0.0027 | 1 (ref) | 0.0647 |
| Z On | 1.55 (1.16 to 2.06) |  | 1.31 (0.98 to 1.75) |  |
| Z Off | 1 (ref) | 0.0100 | 1 (ref) | 0.1610 |
| Z On normal | 1.63 (1.12 to 2.36) |  | 1.30 (0.89 to 1.90) |  |
| Z On high | 1.46 (0.95 to 2.23) |  | 1.37 (0.88 to 2.13) |  |
| Z Linear | 1.28 (1.07 to 1.54) | 0.0075 | 1.20 (0.99 to 1.45) | 0.0644 |
| G Off | 1 (ref) | <0.0001 | 1 (ref) | <0.0001 |
| G On | 5.09 (4.05 to 6.41) |  | 1.81 (1.42 to 2.32) |  |
| **Drug related** |  |  |  |  |
| B Off | 1 (ref) | <0.0001 | 1 (ref) | <0.0001 |
| B On | 2.33 (1.58 to 3.44) |  | 2.90 (1.94 to 4.33) |  |
| B Off | 1 (ref) | <0.0001 | 1 (ref) | <0.0001 |
| B On normal | 1.90 (1.21 to 2.99) |  | 2.48 (1.55 to 3.95) |  |
| B On high | 3.90 (2.20 to 6.90) |  | 4.47 (2.41 to 8.30) |  |
| B Linear | 1.96 (1.50 to 2.55) | <0.0001 | 2.20 (1.67 to 2.89) | <0.0001 |
| Z Off | 1 (ref) | 0.0006 | 1 (ref) | 0.0003 |
| Z On | 2.60 (1.51 to 4.47) |  | 2.77 (1.58 to 4.83) |  |
| Z Off | 1 (ref) | 0.0010 | 1 (ref) | 0.0004 |
| Z On normal | 3.24 (1.69 to 6.21) |  | 3.78 (1.94 to 7.38) |  |
| Z On high | 1.86 (0.76 to 4.57) |  | 1.50 (0.57 to 3.90) |  |
| Z Linear | 1.64 (1.15 to 2.33) | 0.0057 | 1.58 (1.09 to 2.29) | 0.0169 |
| G Off | 1 (ref) | 0.2628 | 1 (ref) | 0.4240 |
| G On | 1.67 (0.68 to 4.09) |  | 1.47 (0.57 to 3.82) |  |
| **Non-drug related** |  |  |  |  |
| B Off | 1 (ref) | 0.6994 | 1 (ref) | 0.5943 |
| B On | 0.94 (0.69 to 1.28) |  | 0.92 (0.67 to 1.26) |  |
| B Off | 1 (ref) | 0.3699 | 1 (ref) | 0.5947 |
| B On normal | 1.04 (0.75 to 1.43) |  | 0.98 (0.70 to 1.36) |  |
| B On high | 0.57 (0.25 to 1.27) |  | 0.65 (0.28 to 1.49) |  |
| B Linear | 0.90 (0.70 to 1.15) | 0.4020 | 0.90 (0.70 to 1.17) | 0.4351 |
| Z Off | 1 (ref) | 0.2846 | 1 (ref) | 0.6267 |
| Z On | 1.28 (0.81 to 2.02) |  | 0.89 (0.56 to 1.42) |  |
| Z Off | 1 (ref) | 0.4438 | 1 (ref) | 0.9055 |
| Z On normal | 1.45 (0.81 to 2.59) |  | 0.97 (0.54 to 1.75) |  |
| Z On high | 1.09 (0.54 to 2.21) |  | 0.85 (0.41 to 1.76) |  |
| Z Linear | 1.12 (0.84 to 1.51) | 0.4353 | 0.93 (0.68 to 1.28) | 0.6711 |
| G Off | 1 (ref) | <0.0001 | 1 (ref) | 0.0005 |
| G On | 6.68 (4.87 to 9.16) |  | 1.82 (1.30 to 2.55) |  |

IRR Incident rate ratio; B benzodiazepines; Z z-drugs; G gabapentoids

*Adjusted for gender, age, year, comorbidity, region, OST type, OST treatment period, and where applicable benzodiazepine, z-drug and gabapentinoid exposure.

Linear trend was applied to ln(IRR). IRR for *on high* was estimated as *on low* squared. The deviation from linearity for adjusted models: Benzodiazepine p=0.6251, 0.7769, 0.5223; Z-drug p=0.4817, 0.0089, 0.8326 for all cause, drug related and non-drug related deaths respectively.

High and normal doses are defined in S2 Table.
