# Supplementary material for: Prescription of benzodiazepines, z-drugs, and gabapentinoids and mortality risk in people receiving opioid agonist treatment: Observational study based on the UK Clinical Practice Research Datalink and Office for National Statistics death records
Source: PLoS Med. 2019 Nov 26;16(11):e1002965. doi: 10.1371/journal.pmed.1002965 (PMC6879111; doi:10.1371/journal.pmed.1002965)
Supplement: S5 Table — (DOCX) [file pmed.1002965.s009.docx]

**Adjusted all cause, drug related and non-drug related incident rate ratios for benzodiazepine, z-drug and gabapentinoid exposure excluding first episode**

|  | All cause mortality | | Drug related mortality | | Non-drug related mortality | |
| --- | --- | --- | --- | --- | --- | --- |
| Exposure | HR (95% CI) | P | HR (95% CI) | P | HR (95% CI) | P |
| B Off | 1 (ref) | 0.0291 | 1 (ref) | <0.0001 | 1 (ref) | 0.8355 |
| B On | 1.37 (1.03 to 1.81) |  | 3.40 (2.00 to 5.79) |  | 1.05 (0.64 to 1.73) |  |
| B Off | 1 (ref) | 0.0504 | 1 (ref) | <0.0001 | 1 (ref) | 0.2535 |
| B On normal | 1.46 (1.08 to 1.99) |  | 3.11 (1.71 to 5.64) |  | 1.34 (0.81 to 2.20) |  |
| B On high | 1.06 (0.58 to 1.91) |  | 4.59 (2.01 to 10.51) |  | 1 (ref) |  |
| B Linear | 1.20 (0.97 to 1.48) | 0.1002 | 2.34 (1.64 to 3.35) | <0.0001 | 0.88 (0.57 to 1.33) | 0.5365 |
| Z Off | 1 (ref) | 0.1337 | 1 (ref) | 0.0386 | 1 (ref) | 0.9150 |
| Z On | 1.40 (0.90 to 2.19) |  | 2.38 (1.05 to 5.40) |  | 1.04 (0.48 to 2.28) |  |
| Z Off | 1 (ref) | 0.2647 | 1 (ref) | 0.0446 | 1 (ref) | 0.9320 |
| Z On normal | 1.39 (0.79 to 2.44) |  | 3.41 (1.30 to 8.97) |  | 1.17 (0.47 to 2.93) |  |
| Z On high | 1.54 (0.76 to 3.13) |  | 1.20 (0.27 to 5.35) |  | 1.14 (0.27 to 4.89) |  |
| Z Linear | 1.28 (0.95 to 1.73) | 0.1099 | 1.46 (0.84 to 2.56) | 0.1804 | 1.10 (0.62 to 1.96) | 0.7335 |
| G Off | 1 (ref) | 0.4417 | 1 (ref) | 0.7794 | 1 (ref) | 0.7181 |
| G On | 1.21 (0.74 to 1.99) |  | 1.19 (0.34 to 4.14) |  | 1.16 (0.53 to 2.54) |  |

HR Hazard ratio; B benzodiazepines; Z z-drugs; G gabapentoids

*Adjusted for gender, year, comorbidity, region, OST type, OST treatment period, and where applicable benzodiazepine, z-drug and gabapentinoid exposure.

High and normal doses are defined in S2 Table
