# Supplementary material for: Prescription of benzodiazepines, z-drugs, and gabapentinoids and mortality risk in people receiving opioid agonist treatment: Observational study based on the UK Clinical Practice Research Datalink and Office for National Statistics death records
Source: PLoS Med. 2019 Nov 26;16(11):e1002965. doi: 10.1371/journal.pmed.1002965 (PMC6879111; doi:10.1371/journal.pmed.1002965)
Supplement: S6 Table — (DOCX) [file pmed.1002965.s010.docx]

**Mortality rates and hazard ratios for Drug-related poisonings by co-prescription exposure to benzodiazepines considering competing risk of death from other causes, z-drugs, or gabapentinoids in people with opioid dependency in primary care**

| **Co-prescription** | **Deaths** | **PY** | **MR** | **Unadjusted** | | **Adjusted*** | | |
| --- | --- | --- | --- | --- | --- | --- | --- | --- |
|  |  |  |  | **HR (95% CI)** | ***P*** | **HR (95% CI)** | ***P*** | |
| Benzodiazepine Off | 74 | 16270 | 0.45 | 1 (ref) | *<0.0001* | 1 (ref) | | *<0.0001* |
| On | 39 | 3679 | 1.06 | 2.27 (1.53, 3.38) |  | 2.86 (2.16, 3.81) | |  |
| Benzodiazepine Off | 74 | 16270 | 0.45 | 1 (ref) | *<0.0001* | 1 (ref) | | *<0.0001* |
| Normal Dose | 25 | 2889 | 0.87 | 1.83 (1.15, 2.90) |  | 2.47 (1.70, 3.58) | |  |
| High Dose | 14 | 790 | 1.77 | 3.86 (2.17, 6.86) |  | 4.04 (2.10, 7.74) | |  |
| *linear effect of dose* | - | - | - | 1.94 (1.47, 2.56) | *<0.0001* | 2.10 (1.65, 2.68) | | *<0.0001* |
| Z-drug off | 98 | 18838 | 0.52 | 1 (ref) | *0.0008* | 1 (ref) | | *<0.0001* |
| On | 15 | 1110 | 1.35 | 2.61 (1.52, 4.51) |  | 2. 82 (1.50, 5.31) | |  |
| Z-drug off | 98 | 18838 | 0.52 | 1 (ref) | *0.0010* | 1 (ref) | | *0.0040* |
| Normal Dose | 10 | 593 | 1.69 | 3.33 (1.73, 6.41) |  | 3.64 (1.56, 8.46) | |  |
| High Dose | 5 | 517 | 0.97 | 1.83 (0.75, 4.51) |  | 1.95 (0.80, 4.72) | |  |
| Gabapentinoid off | 108 | 19410 | 0.56 | 1 (ref) | *0.3200* | 1 (ref) | | *0.5000* |
| On | 5 | 538 | 0.93 | 1.59 (0.64, 3.96) |  | 1.35 (0.57, 2.24) | |  |

*PY – person years follow-up; MR mortality rate (deaths/100 person-years). P (Unadjusted P values testing for differences in mortality rates by co-prescription treatment). High and normal doses are defined in web-table S1. HR Hazard ratio; *Adjusted for sex, year, comorbidity, region, OAT type, OAT treatment period, and where applicable benzodiazepine, z-drug and gabapentinoid exposure*

High and normal doses are defined in S2 Table
