# Supplementary material for: Prescription of benzodiazepines, z-drugs, and gabapentinoids and mortality risk in people receiving opioid agonist treatment: Observational study based on the UK Clinical Practice Research Datalink and Office for National Statistics death records
Source: PLoS Med. 2019 Nov 26;16(11):e1002965. doi: 10.1371/journal.pmed.1002965 (PMC6879111; doi:10.1371/journal.pmed.1002965)
Supplement: Attachment — Submitted filename: PLOS Med_response to reviewers.docx [file pmed.1002965.s011.docx]

Dear Dr Turner

Thankyou for giving us the opportunity to revise and resubmit our manuscript "Prescription of benzodiazepines, z-drugs and gabapentoids and mortality risk in people receiving opioid substitution therapy: observational study based on the UK Clinical Practice Research Datalink and Office of National Statistics death records" (PMEDICINE-D-19-02004). We include a point by point response to both the editorial team and our reviewers below. We hope that revision has improved our manuscript and that it can now be accepted for publication in PLOS Medicine.

Response to general editorial comments

1.) Please ensure all authors complete a contributing author statement and declare conflicts of interest.

    We have requested these of all authors and include those received. We anticipate a complete set of statements very soon. Obtaining this has been complicated by the Summer Vacation season and the general difficulties we have had contacting Dr Colin Steer (see covering letter). Our inability to contact Dr Steer (who undertook all original analyses and who has previously declared no conflict of interest) necessitated us employing another analyst – Dr Rosie Cornish – to complete the additional analyses requested by reviewers. We do not wish to remove Dr Steer as an author as this seems unfair. Under Professor Tilling’s supervision Dr Cornish has both replicated the original analyses and undertaken the additional analyses requested by reviewers. In view of this we ask your permission that she now be included as an author.
2.) Please provide details of your study protocol

    Our project did not have a pre-published protocol. We do however have a full project report, published and open access, which details all our pre-specified hypotheses and is available from the NIHR website at [https://www.journalslibrary.nihr.ac.uk/hsdr/hsdr07030#/abstract](https://www.journalslibrary.nihr.ac.uk/hsdr/hsdr07030#/abstract). In addition our full study protocol detailing all required variables and planned analyses was approved by the CPRD Independent Scientific Advisory Committee (ISAC CPRD Protocol 14_073R2). We have uploaded this.
3.) Please provide details of data availability including any access restrictions.

    Data are available from the CPRD (https://www.cprd.com/home/) for researchers who meet the criteria for access to confidential data.
4.) Please convert your abstract to PLOS Medicine style. The final sentence of the combined "methods and findings" subsection should quote the study's main limitations.

    Thanks, we have now done this (lines 21 – 69 in revised manuscript. We have attempted to include all requested and relevant information whilst adhering to the word limit of 500 words.
5.) Please add an accessible “author summary”

    We have added this (p4, lines 72 – 93 in revised manuscript).
6.) Please add details of your study protocol and pre-specified analyses at the beginning of your methods section.

    We have added this (p6, lines 128 – 132 in revised manuscript).
7.) Please add details of ethical approval to your methods section.

    We have added this (p6, lines 134-139 in revised manuscript).
8.) Please revise your results, discussion and referencing according to house style

    We have done this
9.) Please include a STROBE or RECORD checklist

    We have uploaded a STROBE checklist

Response to comments from the academic editor:

1). The authors' exclusions result in a cohort that is 25% of the original sample. This has implications for generalizability and should be discussed thoughtfully. Second, I am surprised that 50% of the excluded patients were excluded because they were being prescribed buprenorphine or methadone "for pain relief". This proportion seems large. Compared to other large pharmacoepidemiological databases in the US or Europe, is this what one would expect? Or is there just a lot of misclassification going on? This should also be discussed thoughtfully. Sensitivity analyses would also go a long way toward reassuring readers that the estimated associations are robust.

*Response*: Our exclusion criteria were intended to allow a level of certainty that our cohort was restricted to individuals receiving methadone and buprenorphine as Opioid Agonist Therapy as a treatment of Opioid Dependency. This was because all our hypotheses related to the safety of additional prescription of benzodiazepines, z-drugs and gabapentinoids in this population – not in the general population of patients prescribed these medicines. The criteria and their application are clearly described in web figure 1, this approach was also taken to ensure consistency with other work on the relative safety of methadone and buprenorphine in this population that we have published (see refs 7 and 15). It is possible that the stringency of our criteria has meant that some individuals receiving methadone or buprenorphine as OAT have been misclassified as receiving these medicines for pain and excluded from our sample. The main consequence of this will have been a reduction in power. Misclassification in the other direction (i.e. patients receiving these medicines as pain relief misclassified as receiving OAT) is likely to have diluted our ability to detect an adverse effect (as we expect this effect to be seen particularly in OAT patients). It is difficult to see how misclassification would have led to the detection of a spurious adverse effect.

2. Why did treatment episodes include 1 year after the prescription ended? This seems arbitrary and should perhaps be probed with some sensitivity analyses. (The authors select a different cutoff of 14 days for the BZD/z-drug exposure, reasoning that those medications are associated with a "shorter recommended treatment duration". But recommended treatment duration with methadone/buprenorphine is presumably lifelong. So why 28 days?) In addition, I am a little confused about how this worked with patients who had consecutive episodes. If a new episode was defined as a subsequent prescription >28 days after a previous prescription, what if someone had a prescription 45 days after a previous prescription ended? They would still be within the 1-year window, so they would be classified as "in treatment" and this time in treatment would be associated with the first prescription?

*Response*: Rather than being arbitrary, censoring follow-up at one year after the last treatment episode was a decision based on consensus in the research team and again is consistent with the approach in other published work as above. Evidence suggests that cessation of most OAT episodes does not reflect recovery, rather it reflects treatment cessation for other reasons in individuals who are still opioid dependent and continuing to use opioids. We felt it important to consider this phenomenon in our investigation into the safety of additional prescription. However, the longer the period of survival from the last treatment episode the greater the possibility of recovery. As we have shown elsewhere (ref 3), five years after the last treatment episode most surviving patients have indeed recovered and we would not wish to include these patients in analyses considering the safety of additional prescriptions amongst individuals who are still opioid dependent. The choice of one year reflects a balance between these two considerations. Concerns over the inclusion of the period one year after completion of a treatment episode in follow-up only apply to our analyses considering effects of *co-prescription* – this period is not considered in analyses of effects of *concurrent prescription*. Effects of concurrent prescription (table 5) were very similar to effects of co-prescription (these analyses had less power as they were based on fewer events and a shorter follow up period), moreover in our analyses of effects of co-prescription we found no interaction in relation to whether individuals were on or off treatment (table 4 and discussion, second paragraph).

3. The authors do not appear to have made any effort to ensure that treatment episodes (of OST or the co-prescribed BZD/z-drug/gabapentinoids) are "new" treatment episodes.

*Response*: The nature of CPRD data make it impossible for us to know if these are first episodes of OAT treatment. It is also impossible to know whether the first episode of OAT treatment apparent in the data is in an individual who was receiving OAT prior to being observed (for example a transfer from a non-CPRD practice). For this reason we excluded first treatment episodes in a sensitivity analysis. Patterns of association were unchanged.

4. Please justify in the text the use of linear regression (rather than, say, Poisson, negative binomial, two-part models, etc) in the analysis of treatment duration. The authors do not appear to have used robust standard errors so the estimates are unlikely to be robust to a wide range of distributional assumptions.

*Response*: Following a similar point from one of the reviewers, we have now added presentation of the median and IQR to the results, and log transformed the episode duration before analysis. We have used robust standard errors, and accounted for clustering within patient (e.g. some patients may tend to have longer treatment episodes than others).

5. After reading through the methods several times, it is still a little unclear to me exactly what models were used here:

(a) "Where a main effect of co-prescription was suggested by these analyses we investigated whether this effect was modified by an interaction with OST treatment period (i.e. on or off treatment)."

(b) "For benzodiazepine or z-drug dose effects (2 degrees of freedom), a linear trend was also fitted to examine the effect of dose, with “high” doses ascribed based on a daily dose above the maximum recommended in the BNF (see Web table 1)."

(c) "We then assessed the overall effect of concurrent prescription (allowing for any effect on treatment duration) on mortality risk, using Cox proportional hazards survival analysis."

*Response*: we now include details of our models in supplementary text, we hope this provides the clarity requested by the reviewer. We have also removed the text under c) above, “(allowing for any effect on treatment duration)” as this may have given the impression that the model includes treatment duration as a covariate. Rather than this, the meaning we intended to convey was that a proportional hazards survival analysis effectively allows for the longer treatment duration associated with concurrent prescription of benzodiazepines. We have changed this in the paper.

6. The specificity of the findings is unclear. This would be strengthened by the use of an ancillary analysis in which comparable analyses were done with other sedating medications that are commonly co-prescribed in this setting (eg. antidepressants, antipsychotics) as well with other non-sedating medications that are commonly co-prescribed but would not be expected to increase DRP deaths (eg., NSAIDs). This type of analysis is commonly referred to as a "falsification test" (eg., Prasad & Jena, JAMA 2013;309:241-2) or the use of "negative controls" (eg., Arnold et al. Epidemiology 2016;27:637–641)).

*Response*: We felt that the issue of specificity was addressed by our comparison of effects of benzodiazepines, z-drugs and gabapentinoids suggesting an effect on DRP specific to benzodiazepines. We have now undertaken additional analyses examining safety of additional prescription to NSAIDs and antimicrobials. We did not investigate effects of antidepressants and antipsychotics as classic confounding by indication would be expected to generate an association between these and DRP (depression or serious mental illness are both the indications for these medicines and strong risk factors for death by self-poisoning). Results for the analyses investigating effects of concurrently prescribed antimicrobials and NSAIDs on mortality risk are tabulated below. Both co-prescription and concurrent prescription are associated with increased all cause mortality risk (probably reflecting confounding by indication as these medicines will tend to be given to sick people and NSAIDs may also influence CHD risk) however they suggest no consistent effect on risk of DRP. We are happy to report these analyses in response to reviewers’ comments and hope they provide reassurance. Including them in the paper is problematic. They were not pre-specified in our protocol approved by the CPRD ISAC. As part of their review process ISAC often suggest additional analyses but did not suggest these. If we were to include them we would need to request additional ISAC approval.

Additional survival analyses addressing specificity of exposure

Tables A and B for co-prescribed and concurrently prescribed antibiotics

ACM: 12,118 subjects, 655 deaths

DRP/non-DRP: 7112 subjects, 113 deaths from drugs; 282 deaths from not drugs

Table A

| Outcome | Unadjusted |  | Adjusted |  |
| --- | --- | --- | --- | --- |
|  | HR (95% CI) | P | HR (95% CI) | P |
|  |  |  |  |  |
| ACM | 2.73 (1.93, 3.86) | <0.001 | 1.68 (1.10, 2.56) | 0.017 |
| DRP | 1.31 (0.42, 4.14) | 0.64 | 1.37 (0.44, 4.30) | 0.59 |
| Non-DRP | 2.96 (1.78, 4.91) | <0.001 | 1.54 (0.67, 3.52) | 0.31 |

Table B

| Outcome | Unadjusted |  | Adjusted |  |
| --- | --- | --- | --- | --- |
|  | HR (95% CI) | P | HR (95% CI) | P |
|  |  |  |  |  |
| ACM | 1.98 (1.24, 3.17) | 0.004 | 2.51 (1.72, 3.67) | <0.001 |
| DRP | 1.95 (0.62, 6.14) | 0.26 | 3.28 (1.10, 9.81) | 0.034 |
| Non-DRP | 2.06 (1.02, 4.17) | 0.043 | 2.30 (1.10, 4.82) | 0.027 |

Tables C and D for co-prescribed and concurrently prescribed NSAIDs

ACM: 12,118 subjects, 655 deaths

DRP/non-DRP: 7107 subjects, 113 deaths from drugs; 284 deaths from not drugs

Table C

| Outcome | Unadjusted |  | Adjusted |  |
| --- | --- | --- | --- | --- |
|  | HR (95% CI) | P | HR (95% CI) | P |
|  |  |  |  |  |
| ACM | 1.89 (1.47, 2.44) | <0.001 | 1.22 (0.87, 1.71) | 0.25 |
| DRP | 1.35 (0.66, 2.78) | 0.41 | 1.71 (0.71, 4.16) | 0.23 |
| Non-DRP | 2.28 (1.60, 3.27) | <0.001 | 1.09 (0.74, 1.61) | 0.67 |

Table D

| Outcome | Unadjusted |  | Adjusted |  |
| --- | --- | --- | --- | --- |
|  | HR (95% CI) | P | HR (95% CI) | P |
|  |  |  |  |  |
| ACM | 1.20 (0.84, 1.72) | 0.31 | 1.39 (0.88, 2.21) | 0.16 |
| DRP | 1.24 (0.50, 3.03) | 0.64 | 2.45 (0.62, 9.61) | 0.2 |
| Non-DRP | 1.67 (1.04, 2.69) | 0.035 | 1.64 (0.93, 2.91) | 0.09 |

MINOR COMMENTS:

7. I would recommend using the term "opioid agonist treatment", or some other non-stigmatizing term, rather than "opioid substitution therapy". See Wakeman SE. J Addict Med 2017;11: 1–2.

*Response*: We are happy to adopt this suggestion.

8. The authors indicate they studied 10 benzodiazepines, 3 z-drugs, and 2 gabapentinoids. These should be named.

*Response*: These drugs are named in web table 1.

9. I understand that the CRPD initial cohort received a prescription between 1st January 1998 and 31st July 2014. In the Results section, the authors should provide the respective dates for the final analytic sample.

*Response*: These were also the dates for the final analytic sample.

10. More detail should be provided in the summary statistics paragraph: "These data contained 29,540 treatment episodes: 17,391 methadone (median treatment duration, XXXX; range, XX-YY; interquartile range, XX-YY), 9180 buprenorphine (median treatment duration, XXXX; range, XX-YY; interquartile range, XX-YY) and 2969 episodes relating to more than one drug. Total follow-up time was 36,126 person years during which there were 17,139 episodes of benzodiazepine treatment, XXXX episodes of z-drug treatment, and YYYY episodes of gabapentinoid treatment. Benzodiazepines were prescribed during 10,022 OST episodes in 7059 patients. Z-drugs were prescribed during 4279 OST episodes in 2822 patients. Gabapentoids were prescribed during 1827 OST episodes in 1281 patients."

*Response*: we have now provided this detail (see table 1 and Results first paragraph).

11. The authors indicate "mean follow-up per patient of 3.4 years" but this should be expressed as "median (IQR; range)".

*Response*: These have now been added to table 1.

12. Throughout the manuscript the authors refer to "gabapentoids". These should be replaced with "gabapentinoid".

*Response:* We used the terminology recommended by our UK Pharmacology colleagues. We are happy to use the alternative terminology suggested if this has greater international recognition.

13. The literature review is incomplete. Several studies of co-prescribing in this area have been published, but the authors neither cite nor interact meaningfully with this literature (eg., discussing the strengths of their study relative to the others).

Gomes T et al. PLoS Med. 2017 Oct 3;14(10):e1002396.
Gomes T et al. Ann Intern Med 2018;169:732-4
Johansen. JAMA Intern Med. 2018 Feb; 178(2): 292–294.
Peckham et al. Drug Saf 2018;41(2):213-228
Montastruc et al. JAMA. 2018;320(20):2149-2151.

*Response*: Thank you for suggesting these additional papers that we now cite and discuss briefly (Discussion fifth paragraph, refs 23 – 27). We feel that none of them consider precisely the same question as our study (safety of additional prescription of benzodiazepines, z-drugs or gabapentinoids in OAT patients) however we agree they are relevant and apologise for their omission previously. Two derive from a Canadian case-control study looking at risk of DRP with gabapentinoids in all patients prescribed opioids other than for palliative care or cancer. Results are similar to our own. One describes increase in use of gabapentinoids in the US between 2002 and 2015 and also highlight increases in patients prescribed both gabapentinoids, opioids and benzodiazepines. One describes pharmacovigilance around gabapentinoids in the US. The final paper uses data from the CPRD to describe increases in gabapentinoid use in the UK between 1993 and 2017.

Comments from the reviewers:

*** Reviewer #1:

The authors present a cohort study using administrative data from 1998 - 2014 from the United Kingdom examining over 12,000 patients receiving opioid substitution treatment (OST) and their duration of OST use and mortality in relation to use of benzodiazepines, z-drugs (zaleplon, zolpidem, and zopiclone), and gabapentoids. The findings of the study most strongly suggest an increased duration of OST associated with concurrent benzodiazepine use but also a significantly increased risk of mortality relative to non-use. A few comments and questions are noted below:

1.      It's unclear whether the cohort was a 'new user' cohort (i.e. relatively new use of OST). If it was a new user cohort, please include the definition of 'new user'. If not, would it be possible to provide a sense of how long they had being OST prior to the cohort entry / start date? As the authors may be aware, prevalent user designs may be subject to various sources of bias (<https://www.ncbi.nlm.nih.gov/pubmed/14585769>). On a related note, how was the cohort entry date / start date defined? Was it the first instance of OST use observed during the study period? Please explicitly define.

*Response*: Please see our response 3 above. As previously noted, it is impossible to identify new users in the CPRD (we can identify first complete observed episodes). The concerns around “prevalent users” relate to survival bias which would tend to dilute apparent adverse effects of additional prescription and effects of the treatment itself on confounding factors. Again we find it difficult to see how bias of the second type could generate apparent yet spurious adverse effects of additional prescription in the patterns we observed. We are happy to accept (and we emphasise in the paper) the general point that the possibility of residual confounding cannot be excluded.

2.      For the exposures of interest, these drugs are often used on an as needed (prn) basis and 14 days was assumed to be the minimum gap between prescriptions for purposes of estimating use (page 8). Was this an arbitrary decision? How was occasional use handled (e.g. some people may spread the use of 30 pills of benzodiazepines over 90 days)?

*Response*: This is a reasonable point, the rationale for choosing 14 days was not arbitrary and was reached based on consensus amongst clinical members of the team considering current recommendations around prescription duration of benzodiazepines and z-drugs. We cannot account for the fact that some patients may have used these medicines intermittently on an as required basis. The most likely consequence of this would have been to dilute any apparent adverse effects.

3.      Was there a single primary endpoint of interest? There were numerous exposures, definitions of exposure duration, and outcomes. As a result, quite a few statistical tests were conducted. How was the issue of multiple hypothesis testing handled?

*Response*: All our hypotheses were pre-specified and in relation to each we considered magnitude of the effect, evidence of dose-response associations (where possible) and evidence against the nul hypothesis of no effect. This approach is not driven by consideration of arbitrary p value threshold therefore “correction” of these for multiple testing is irrelevant (see Sterne JA, Smith GD. Sifting the evidence-what's wrong with significance tests? [BMJ.](https://www.ncbi.nlm.nih.gov/pubmed/11159626) 2001 Jan 27;322(7280):226-31.).

4.      Were comorbidities assessed prior to cohort entry (i.e. the start date) or were 'new' comorbidities identified during follow-up included (page 8)? The use of time varying covariates in this regard is a bit confusing. Please explain.

*Response*: It was impossible to assess co-morbidity prior to cohort entry. These are all “prevalent” comorbidities which have the potential to vary over time – hence the use of a time varying covariate.

5.      The definition of death involves multiple data sources (page 9). Has the outlined approach to defining death been validated? If so, how accurate is death using the approaches taken? Also, have there been any validation studies of the outcome of drug-related death poisoning?

*Response*: We use the approach to defining DRP adopted by the UK Office for National Statistics <https://www.ons.gov.uk/peoplepopulationandcommunity/birthsdeathsandmarriages/deaths/datasets/deathsrelatedtodrugpoisoningbyselectedsubstances>. The CPRD dataset is one of the most comprehensive prospective primary care databases, the validity of which has previously been reviewed elsewhere (e.g. see ref 14, and Khan NF, Harrison SE, Rose PW. Validity of diagnostic coding within the General Practice Research Database: a systematic review. Br J Gen Pract. 2010;60(572):e128–e36. pmid:20202356 and Herrett E, Thomas SL, Schoonen WM, Smeeth L, Hall AJ. Validation and validity of diagnoses in the General Practice Research Database: a systematic review. Br J Clin Pharmacol. 2010;69(1):4–14. pmid:20078607).

6.      Linear regression was used to examine the association between concurrent use of an exposure of interest and OST. Was a time-to-event analysis such as a survival model considered for this assessment? Were competing risks considered?

*Response:* Thank you for this point which was also made by another reviewer. Table 5 presents results of a survival analysis. We have now undertaken an additional competing risks analysis, the results of this are included in an appendix (web table 6), they are essentially unchanged.

7.      It would be helpful to more fully describe how selection bias was handled in this study. For example, patients not using a benzodiazepine may be considerably different than those who use a benzodiazepine. Such information has not been provided. Use of time-varying covariates in a survival model does not obviate selection bias. Further, timing of as needed drugs such as benzodiazepines may reflect critical events in a patient's life that lead to the use of such medications. The critical events may have a stronger effect on the risk of mortality than the actual exposure being assessed (i.e. the exposure to a drug such as a benzodiazepine at a particular time may mask a critical event that may not be captured by the available data). It would be helpful for the authors to highlight how such temporal confounding was handled, if at all.

*Response*: The fundamental issue here is the possibility of residual confounding either by indication or by other unmeasured covariates. We describe our efforts to overcome confounding but readily acknowledge the possibility that residual confounding remains. Ultimately, as in all observational research, readers must make their own judgements around causal inference.

*** Reviewer #2:

This is a well-conducted epidemiological study on the associations between prescription of benzodiazepines, z-drugs and gabapentoids and mortality risk in people receiving opioid substitution therapy based on UK's CPRD primary care database and death records from the Office of National Statistics. The study design, datasets, statistical methods and analyses, presentations (tables and figures) and interpretations of results are mostly adequate and of a good standard. However, there are still a few important statistical issues needing attention.

1) The key conclusion of the study is that Co-prescription of benzodiazepine is associated with a three-fold increased risk of DRP (drug-related poisoning mortality) as shown in table 3. This was done by the Cox model.  However, in this case all cause mortality becomes a competing risk that could potentially hinder or modify the chance of DRP, therefore it would be appropriate to estimate the risk of DRP within the competing risk framework. Unfortunately the authors didn't address this issue in the paper.

*Response*: Thanks, we acknowledge this point and have conducted a competing risks analysis as described above. As previously noted results were essentially unchanged.

2) Table 2 on treatment durations. In the second column, median duration was presented which suggested that the distributions of the duration were not normally distributed. Is this right? If so, then these durations as outcome should be transformed (e.g., log-transformation) into normal variables before they can be used in linear regressions. Can authors check, report and correct as appropriate?

*Response*: As pointed out, the duration is highly positively skewed, and thus we have now provided the IQR as well as the median, and used the log transformation before analysis.

(see table 2b)

3) Table 1. Follow-ups (years) should be summarised as median and IQR rather than mean and SD as they are skewed data.

*Response*: We have now done this (see revised table 1)

*** Reviewer #3:

This paper is important as it addresses a question that has, and should be, high on the agenda for policy makers and clinical practitioners managing people who have used drugs.
It has been difficult in the past to unravel the interactions of drugs and their, often fatal, effects. Using administrative data is an innovative way to provide some clarity and although the authors acknowledge the incomplete nature of their enquiry into this complex area they have opened up some very important leads and articulated the research questions that need to be answered.

1.	Are benzodiazepines prescribed because methadone doses are too low? This is a common problem in clinical practice and diazepam particularly, but also other sedatives such as Z drugs, are prescribed or obtained illegally to supplement the effects of inadequate opiate substitute treatment.

*Response:* This is an interesting hypothesis but not one that we pre-specified or that we are able to consider in our data. We have no way of knowing whether participants obtain illicit benzodiazepines (or opioids). We agree that it is possible that they do. The likely result is random misclassification of exposure leading to dilution of apparent effects.

2.	People on <20mg methadone excluded. This clearly is a dose way below the recognized therapeutic level but isn't 30 or 40mg in the same range?

*Response*: We have discussed our relatively stringent exclusion criteria alongside the rationale for these (including consistency with other published studies) above. We have also discussed bias that may have resulted from any misclassification.

3.	Are these patients using non prescribed BDZs. How is it known that the BDZ present at death isn't non prescribed?

*Response:* As discussed above we have no information regarding use of illicit medicines.

4.	Mortality showed regional variation, is there any explanation for this variation and is it associated with coprescribing?

*Response*: We think that the cause of regional variation is likely to be multifactorial, it was not associated with co-prescribing.

5.	With evidence of a dose response effect can it be made clearer how strong this was? Is it not true that there would be and expectation that it would be significant?

*Response*: Dose response relations and the uncertainty estimates associated with them are shown in table 3.

6.	Why not include other drugs with an equal potential for additional respiratory depression such as SSRIs and those with QRS complex impairment?

*Response:* We now further explore the question of specificity by considering effects of additional prescription of NSAIDs and antimicrobials. We explain why we did not consider SSRIs above.

7.	The former are widely used to supplement opiate substitute treatment and to complement the management of the symptomatology around a lifestyle of opiate use.
What is the mechanism for association of co prescription of benzodiazepines and methadone and DRP? Is it possible to comment or even speculate on whether or not this is cumulative respiratory depression or if, as had been suggested, that co prescribed drugs has a behavioural effect in inducing confusion/recklessness or misjudgement of doses?
We already know that deaths are associated with multiple drugs at toxicology but the causation is complex and less well understood.

8.	Research is needed into the reasons why overdose can occur when it hasn't before and the role of other factors such as COPD and GI (liver primarily) disorders, reference for example; Lu Gao J. Roy Robertson Sheila M. Bird Non drug-related and opioid-specific causes of 3262 deaths in Scotland's methadone-prescription clients, 2009-2015 Drug and Alcohol Dependence Volume 197, 1 April 2019, Pages 262-270

*Response to 7 and 8*: As the reviewer correctly states there are several possible mechanisms through which additional prescription of benzodiazepines could causally influence risk of death from drug related poisoning and we cite animal evidence for the potentiation of respiratory depression in the penultimate paragraph of our discussion. None of these mechanisms can be tested in our study.

9.	Is this subset a higher risk group with different characteristics? It is recognised that drug users are not a homogeneous group with varying behaviour patterns suggesting an increased risk

*Response*: Again this relates to the possibility of residual confounding that we have discussed above.

10.	The conclusion that benzodiazepines should not be coprescribed is a sweeping statement based on results that don't necessarily take into account the best intentions of clinical services that are trying to balance the needs for symptomatic treatment of a distressed caseload. Avoiding one drug (such as diazepam in this case) often leads prescribers to look for an alternative, which may be equally toxic.

The other, perhaps more recent, problem is the escalation in availability of synthetic or illegally sourced benzodiazepines. Supplies of etizolam and alprazolam have distorted the marked and caused enormous self-medication problems leading to increased risk of death. One response to this has been to accommodate these needs by prescribing benzodiazepines. Perhaps the underlying problem is inadequate dosing of methadone or buprenorphine and it might be useful to say this.

*Response*: We address the point about possible “inadequate” dosing of OAT above. Other than through restricting our sample to individuals receiving methadone or buprenorphine at a dose felt likely (by consensus) to represent OAT we cannot test (and did not prespecify) this hypothesis. In particular, the dose of OAT an individual is prescribed does not allow inference of “adequacy” for that individual as this issue depends on both tolerance and individual expectations and needs. Because this question does not relate to a prespecified hypothesis and cannot be informed by our data it seems inappropriate to suggest that the “underlying problem” is inadequate dosing of OAT.

***
