# Supplementary material for: Prescription of benzodiazepines, z-drugs, and gabapentinoids and mortality risk in people receiving opioid agonist treatment: Observational study based on the UK Clinical Practice Research Datalink and Office for National Statistics death records
Source: PLoS Med. 2019 Nov 26;16(11):e1002965. doi: 10.1371/journal.pmed.1002965 (PMC6879111; doi:10.1371/journal.pmed.1002965)
Supplement: Attachment — Submitted filename: response to reviewers PLOS MED 101019.docx [file pmed.1002965.s012.docx]

Requests from Editors:
Abstract – where there are 95% values for quantifiable data, please also add p values (also in main text and tables, where necessary). Even though the abstract does not exceed the requested 500 words, that doesn’t need to be a target and I find it quite long and wonder if it could be trimmed for conciseness?

*Thank you we have done this*

Abstract – as this isn’t a trial, please be cautious to avoid causal language…for example in the abstract “In this study, co-prescription of benzodiazepine was associated with increased risk of DRP in opioid dependent individuals both on and off OAT. Co-prescription of z-drugs and gabapentinoids was also associated with increased mortality risk, however evidence for causality was less strong.” Please avoid the word evidence and also vague language…an association is either significant or not and if not, remove – or say no association. Also line 312 (causal effect).

*Thank you we have made these changes throughout the paper. We sought clarification from the editorial office regarding use of the term “statistically significant”. Normally we avoid use of this language however we were advised of your preference for it. We have complied with this advice. We also note that in our submission we have included some p values with a greater degree of precision than would normally be the convention. This was an oversight on our part though was not mentioned by the editors. At present we have not corrected it.*

Author summary – thanks you for adding this; it needs to be in a bullet point format.

*Thankyou we have added bullet points*.

Line 259 – “was not strongly associated with age” – please be specific in terms of significance of association – it is, or isn’t.

*Thank you we have changed this as above.*

Line 423 (should) please say ‘may be ‘ and would bit be more accurate to say applicable in other countries instead of externally valid?

Thank you we have changed this as advised

Line 428 (Our findings have clear implications for clinical policy) – please say ‘may have’ and remove ‘clear’

*Thank you we have changed this as advised*

Line 442 (Our data did not  provide strong evidence that co-prescription of gabapentinoids to patients receiving OAT causes increased risk of DRP) – please be clear – it did, or didn’t…

*Thank you we have changed this to*

*“Our data did not show a significant association between co-prescription of gabapentinoids to patients receiving OAT and increased risk of DRP. These analyses were under-powered.” Lines 446 to 448.*

STROBE – thanks for providing. Instead of METHODS THIRD TO FIFTH PARAGRAPH (for example), please say Methods Paragraphs 3-5 and adjust throughout. Also please remove the word ‘Done’.

*Thank you we have made these changes.*

Reviewer #1: While the authors have dutifully responded to the many comments of the editors and reviewers, I am not convinced that the validity of the study findings are high enough to warrant publication in PLOS Medicine. Several outstanding issues have either not been addressed adequately or simply cannot be addressed well given the nature of the study design:
1. Generalizability: The academic editor raised the issue of a very high exclusion rate (i.e. a study cohort that is 25% of the original sample). While the authors provide some explanations for the exclusions, the level of misclassification is unclear and a validated approach to arriving at the study sample would have been extremely helpful. Simply accepting a possibly high level of misclassification doesn't seem adequate. Some more 'homework' needs to be done in this regard.

*We apologise if our previous explanation was unclear. The inclusion criteria we used were intended to minimise the risk of misclassification bias (including people who were prescribed opioids for pain rather than as OAT) based on prescription and diagnostic data recorded by the practices. This is the accepted way of defining cohorts or “risk sets” using primary care databases. We have used this approach in other studies that have been published in leading journals (for example see references 7 and 15 in the paper). We hope that this provides the reassurance that the approach is accepted as valid sought by the reviewer. As we acknowledged in our previous response it is possible that the stringency of our criteria has meant that some individuals receiving methadone or buprenorphine as OAT have been misclassified because they were prescribed low dose of methadone or buprenorphine and had insufficient diagnosis information. The main consequence of this will have been a reduction in power. Misclassification in the other direction (i.e. patients receiving these medicines as pain relief misclassified as receiving OAT) is far less likely and if it occurred would dilute our ability to detect an adverse effect (as we expect this effect to be seen particularly in OAT patients). It is difficult to see how misclassification would have led to the detection of a spurious adverse effect. Since this point was originally raised by the academic editor and was not revisited in the requests from editors, we hope that this indicates that the editors are satisfied with our response. With the greatest respect we don’t understand what additional “homework” we might usefully do in this regard.*

2. Establishing a 'new user' cohort: New user designs are known to minimize bias, however the authors acknowledge that this study design is virtually impossible with the data sources available. It may be that 'chronic' OAT users may be more likely to use one of the study drugs than another. The use of time-dependent exposure assessment may help to mitigate some of the potential biases here, but it still may be problematic.

*The reviewer is reiterating the argument that longitudinal data where the point where any recipient of OAT receives their first ever prescription of OAT is apparent would reduce some potential biases related to survival and chronicity of opioid dependence. We agree with this argument. However, as the reviewer acknowledges such a cohort cannot be constructed from administrative databases like CPRD. Not all English practices contribute to CPRD and CPRD coverage does not extend across the complete life course of all the individuals it covers. Because of this there will inevitably be periods of the life course of some participants that are unobserved. Moreover, administrative data do not allow examination of periods of opioid dependency prior to any OAT treatment nor do they allow assessment of effects of non-prescribed medicines. All these considerations apply to most of the hundreds of published studies based on CPRD and other administrative databases. We don’t think (and we doubt if the reviewer thinks) that this invalidates the results of these studies – particularly where they relate to important clinical questions not readily amenable to study in other ways.*

3. Temporal confounding and specificity of findings: Perhaps most concerning is the possibility of temporal confounding as it relates to protopathic bias. The issue here is that the initiation of a drug (e.g. a benzodiazepine) may 'unmask' an underlying problem (either related or unrelated to the drug being initiated) that then leads to an outcome such as death or overdose. The use of 'falsification tests' or 'negative controls', as suggested by the academic editor may help understand this issue better. The use of antidepressants / antipsychotics and NSAIDs was suggested by the academic reviewer. The authors did not examine the effects of antidepressants / antipsychotics since they felt there may be considerable confounding by indication. I suppose, perhaps to a lesser extent, the same argument could be made for benzodiazepines. The authors did, however conduct such analyses using antimicrobials and NSAIDs. The findings highlight the issue of bias as statistically significant associations between antimicrobials and several of the outcomes of interest were observed. The risk estimates for the association between NSAIDs and the outcomes of interest were also elevated but not statistically significant. These findings raise serious concerns about the validity of the observed association between benzodiazepines and the selected outcomes. The authors seem to dismiss the associations between antimicrobials and the selected outcomes by suggesting these are driven by confounding by indication and fail to consider that this may also be what's driving the observed associations between benzodiazepines and the selected outcomes.

For the above key reasons, I question the validity of the findings of this study.

*We accept that the greatest threat to causal inference in observational pharmaco-epidemiological studies based on administrative databases is confounding by indication i.e. an observed effect that is caused by a characteristic that leads an individual to be more likely to be prescribed a medicine (an indication) rather than the medicine itself. We also accept, and have made this clear in our paper, the reviewer’s point that the effect of benzodiazepines on increased risk of Drug Related Poisoning (DRP), reported in our paper may arise through confounding by indication. Using the criteria to help guide causal inference originally proposed by Bradford-Hill we made the point that the association between co-prescribed and concurrently prescribed benzodiazepine and increased risk of DRP seemed reasonably specific. That is, it wasn’t as clearly apparent in relation to other mortality classes. Because of the most likely mechanisms (either potentiation of respiratory depression or impaired judgement around safe use) we would expect a causal relation between a co-prescribed sedative medicine and increased mortality risk to be specific to DRP. We suggest that this is further, albeit circumstantial, evidence of a causal relation. In contrast, Z-drugs were associated with both DRP and All-Cause Mortality (ACM) whereas gabapentinoids were associated with ACM but not DRP. The reviewers invited us to consider this question of specificity further by examining effects of other drug classes such as NSAIDs and antimicrobials. Since we had these data available (though do not currently have permission to publish analyses based on them) we were pleased to do this. We thought that the results of these additional analyses provided further circumstantial evidence in favour of our argument for causality in that the strongest and most consistent association present in them is an association with ACM. We argue that this would be expected based on confounding by indication and known effects of these medicines – both will tend to be given to sicker individuals and NSAIDs are known to increase risk of cardiovascular mortality. Reviewer 1 however disagrees and highlights instead that the adjusted association between concurrently prescribed antimicrobials and DRP is conventionally significant. This is not true of the unadjusted estimate nor of the associations between co-prescribed antimicrobials and DRP. We think that it is worth making the point that these analyses were done as quickly as possible (given editorial deadlines) by an analyst new to the project (Dr Rosie Cornish). We are happy with the provenance of the additional analyses however had we had more time we would have explored them further, testing all assumptions and particularly focusing on the surprising adjusted association between concurrently prescribed antimicrobials and DRP. We suggest that it wouldn’t be helpful for us to do this. It would necessitate us seeking additional approval from the CPRD ISAC which would introduce delay. Moreover, reviewer 1’s central point is that the possibility of confounding by indication cannot be dismissed. We agree and have said that in the paper.*

*We respectfully disagree that this point means that our findings should not be published. The same point could be made about many papers published in the Lancet, the BMJ indeed any journal that has published analyses based on prescribing data.*

Reviewer #2: Thanks authors for their effort to improve the manuscript. The response and revision are mostly satisfactory. However, still one minor issue remaining. In the revised table 1, could authors please remove the row with Mean follow-up years (SD)? For skewed data, it doesn't make sense and is inadequate to use mean and SD. Median and IQR are sufficient and adequate in this case.

*Thank you, we have made this change*


Reviewer #3: I am happy that the authors have considered and responded to my suggestions and concerns. I understand that many of my comments were outwit the range of the study and my expectations were, for that reason, unrealistic. I do think that more research is required to illuminate some of the unanswered questions but that is clearly beyond the scope of this study.
I am still worried about the statement in the abstract and text that prescribers should avoid prescribing benzodiazepines completely. This is an unrealistic expectation in an era of enhanced Harm Reduction when availability of illegal benzodiazepines and self medication is at an all time high. I accept that BDZs and other coprescribed drugs may increase the risk of death but there must be a balance of risks present (as there is with many drugs that are commonly prescribed in patients with serious disease). For an influential group to make a uncompromising statement is likely to cause  unintended consequences. Guidelines will cite it and conclude that prescribing any BDZ is outwit normal practice and any strategy to reduce harm by prescribing a (relatively safer) benzodiazepine will be inhibited.
Could the authors draw attention to the risk they have identified and suggest less binary solution?

*We thank reviewer 3 for these thoughtful comments. Considering them (alongside reviewer 1’s enduring concerns regarding causality) we have toned down our suggestions around implications for clinical practice to suggest caution is warranted around co-prescription rather than that benzodiazepines should *never* be co-prescribed. The relevant part of our conclusions now reads*

*“Prescription of benzodiazepines to opioid dependent patients during OAT or whilst still using illicit opioids should generally be avoided other than when clinical judgement suggests that the benefits of this are likely to be greater than the harms. Clinical guidelines should emphasise this point.”*

*Lines 432 - 435*

*As noted above we continue to acknowledge in the paper that the possibility of residual confounding can never be dismissed in studies of this kind.*
